# Synergistic targeting of *BRCA1* mutated breast cancers with PARP and CDK2 inhibition

**DOI:** 10.1038/s41523-021-00312-x

**Published:** 2021-08-31

**Authors:** Diar Aziz, Neil Portman, Kristine J. Fernandez, Christine Lee, Sarah Alexandrou, Alba Llop-Guevara, Zoe Phan, Aliza Yong, Ashleigh Wilkinson, C. Marcelo Sergio, Danielle Ferraro, Dariush Etemadmoghadam, David D. Bowtell, kConFab Investigators, Violeta Serra, Paul Waring, Elgene Lim, C. Elizabeth Caldon

**Affiliations:** 1 Centre for Translational Pathology, Department of Pathology and Department of Surgery, University of Melbourne, Parkville, VIC Australia; 2 Peter MacCallum Cancer Institute, Victorian Comprehensive Cancer Centre, Parkville, VIC Australia; 3 Department of Surgery, University of Melbourne, Parkville, VIC Australia; 4 Pathology Department, College of Medicine, University of Mosul, Mosul, Iraq; 5 The Kinghorn Cancer Centre, Garvan Institute of Medical Research, Sydney, NSW Australia; 6 St. Vincent’s Clinical School, Faculty of Medicine, UNSW Sydney, Sydney, NSW Australia; 7 Experimental Therapeutics Group, Vall d’Hebron Institute of Oncology, Barcelona, Spain; 8 Sir Peter MacCallum Department of Oncology, The University of Melbourne, Parkville, VIC Australia

**Keywords:** Predictive markers, Breast cancer

## Abstract

Basal-like breast cancers (BLBC) are aggressive breast cancers that respond poorly to targeted therapies and chemotherapies. In order to define therapeutically targetable subsets of BLBC we examined two markers: cyclin E1 and *BRCA1* loss. In high grade serous ovarian cancer (HGSOC) these markers are mutually exclusive, and define therapeutic subsets. We tested the same hypothesis for BLBC. Using a BLBC cohort enriched for *BRCA1* loss, we identified convergence between *BRCA1* loss and high cyclin E1 protein expression, in contrast to HGSOC in which *CCNE1* amplification drives increased cyclin E1. In cell lines, *BRCA1* loss was associated with stabilized cyclin E1 during the cell cycle, and *BRCA1* siRNA led to increased cyclin E1 in association with reduced phospho-cyclin E1 T62. Mutation of cyclin E1 T62 to alanine increased cyclin E1 stability. We showed that tumors with high cyclin E1/*BRCA1* mutation in the BLBC cohort also had decreased phospho-T62, supporting this hypothesis. Since cyclin E1/CDK2 protects cells from DNA damage and cyclin E1 is elevated in *BRCA1* mutant cancers, we hypothesized that CDK2 inhibition would sensitize these cancers to PARP inhibition. CDK2 inhibition induced DNA damage and synergized with PARP inhibitors to reduce cell viability in cell lines with homologous recombination deficiency, including *BRCA1* mutated cell lines. Treatment of *BRCA1* mutant BLBC patient-derived xenograft models with combination PARP and CDK2 inhibition led to tumor regression and increased survival. We conclude that *BRCA1* status and high cyclin E1 have potential as predictive biomarkers to dictate the therapeutic use of combination CDK inhibitors/PARP inhibitors in BLBC.

## Introduction

Breast cancer with *BRCA1* mutation most often manifests as basal-like breast cancer (BLBC)^1^, which presents difficulties for treatment as these cancers present at an earlier age, at a high grade, and with greater tumor burden. With the exception of immunotherapy/chemotherapy combination for PD-L1 positive patients^2^, there are currently no targeted therapies routinely used to treat BLBC^3^.

*BRCA1* is a central component of the homologous recombination DNA repair pathway, and its loss results in compromised DNA damage repair^4^. Alterations to *BRCA1* are important founder mutations for breast cancer^5^, and notably, more than 70% of *BRCA1* mutation carriers develop early-onset BLBC based on gene expression profiling^6^. *BRCA1* mutation directly drives the basal phenotype, and mice with *Tp53* and *Brca1* deletion develop mammary tumors with basal-like characteristics^7^ while intact *Brca1* represses the transcription of basal cytokeratins^8^.

A previous report identified that BLBCs from patients with germline *BRCA1* mutation was associated with high cyclin E1 protein expression^9^. Cyclin E1 is a cell cycle regulatory protein that activates cyclin-dependent kinase 2 (CDK2), and whose gain can promote both increased proliferation and genomic instability in cancer cells, and is frequently elevated in BLBC^10^. Perplexingly, in high grade serous ovarian cancer (HGSOC) cyclin E1 amplification and *BRCA1/2* mutation are mutually exclusive, presumably because both aberrations drive genomic instability and together they precipitate lethal genomic damage^11–13^.

We recently described two subsets of HGSOC, one where cyclin E1 gene amplification and *BRCA1* mutation were mutually exclusive, and another where high cyclin E1 protein expression was due to post–transcriptional deregulation rather than gene amplification, and was often concurrent with *BRCA1/2* mutation^13^. Cyclin E1 protein stability is regulated by a multi-step process of specific phosphorylation and ubiquitination, leading to its cyclic expression and turnover^14^. Key regulators in the turnover of cyclin E1, such as the ubiquitin ligase component FBXW7 and the deubiquitinase USP28, are frequently dysregulated in cancer^14–16^ leading to altered stability of the cyclin E1 protein.

In this study, we examined whether *BRCA1* loss and cyclin E1 gain occurred concurrently or independently in breast cancer. We also explored the mechanisms underpinning high cyclin E1 expression in *BRCA1* mutated breast cancer including gene amplification and protein stability. Finally, we tested the hypothesis that disruption of cyclin E1/CDK2 function would sensitize *BRCA1*-mutant cells to PARP inhibition by enhancing synthetic lethality.

## Results

### *BRCA1* inactivation associates with high cyclin E1 expression in breast cancer

**Fig. 1:**
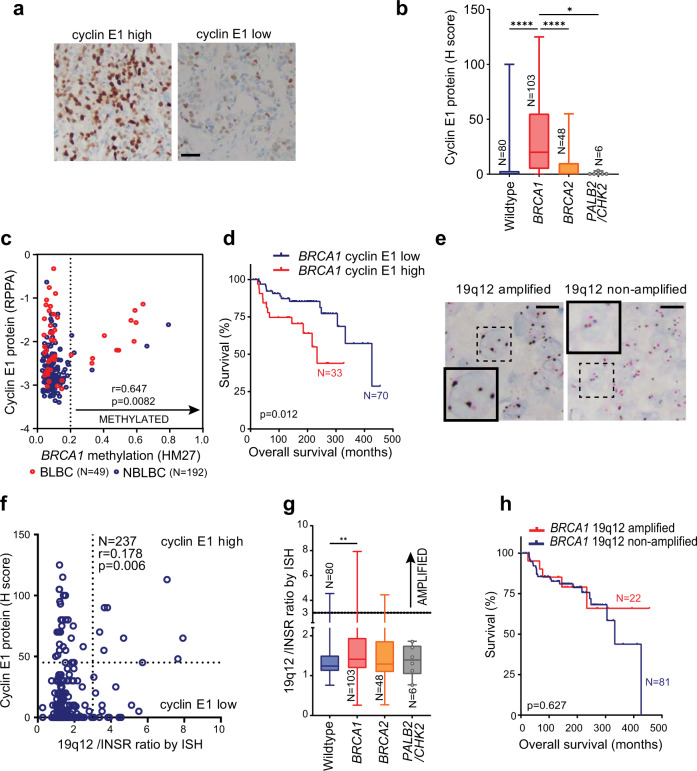
Cyclin E1 is elevated in *BRCA1* deficient cancers, and predicts poor prognosis. **a** Microscope images of high and low expression of cyclin E1 (IHC). Scale bar is 50 µm. **b** Cyclin E1 protein expression (H score) in wildtype, *BRCA1*, *BRCA2,* and *PALB2*/*CHEK2* mutated cancers, analysis by one-way ANOVA; *p < 0.05; ****p < 0.0001. Box and whisker plots show error bars of minimum to maximum, where the box extends from the 25th to the 75th percentile, and the line in the middle of the box indicates the median. **c** Scatter plot of TCGA breast cancer cohort cyclin E1 protein expression (RPPA) versus *BRCA1* methylation by HM27 array. Dashed line indicates cut-off between methylated and non-methylated. Correlation analysis of cyclin E1 protein and *BRCA1* methylation performed across the methylated subset, *r* = Spearman coefficient. **d** Kaplan–Meier curves of overall survival in the KConfab cohort comparing *BRCA1* mutated cyclin E1 high cases versus *BRCA1* mutated cyclin E1 low cases. **e** Microscope images of 19q12 non-amplified and 19q12 amplified breast cancer cases (ISH); inset shows representative example of each. Scale bar is 20 µm. **f** Scatter plot of cyclin E1 protein expression versus *CCNE1* (19q12/INSR ratio) amplification status in the KConfab cohort. *r* = Spearman coefficient. **g** 19q12/INSR ratio (ISH) cases compared to each of wild type, *BRCA1*, *BRCA2, PALB2*/*CHK2* mutated cancers in the KConfab Cohort, analyzed by one-way ANOVA; **p < 0.01. Box and whisker plots show error bars of minimum to maximum, where the box extends from the 25th to the 75th percentile, and the line in the middle of the box indicates the median. **h** Kaplan–Meier curves of overall survival of *BRCA1* mutated breast cancer comparing 19q12 amplified and non-amplified subsets.

We examined the KConFab cohort, which is enriched for familial cancer mutations, for co-occurrence of germline *BRCA1* mutation and high cyclin E1 expression. First, we examined cyclin E1 expression by immunohistochemistry (IHC) (Fig. 1a). High cyclin E1 expression was defined as an H score cut-off of ≥45 based on the overall distribution of cyclin E1 expression (Supplementary Fig. 1a), and previous reports^10,17^. This cut-off was also associated with patient outcomes (minimal *P* value, Supplementary Table 1). Overall, germline *BRCA1* mutated cancers had significantly higher cyclin E1 protein than the *BRCA1* wildtype cases, and tumors with other breast cancer associated germline mutations (*BRCA2*, *PALB2*, or *CHEK2)* (Fig. 1b). Moreover, a significantly larger proportion of germline *BRCA1* mutant cases (80.6%) had detectable cyclin E1 protein (83/103) compared to only 35.1% of *BRCA1* wildtype tumors (47/134) (*P* < 0.0001, Fisher Exact test).

Notably, seven of the germline *BRCA1* wildtype tumors had high cyclin E1. We hypothesized that these may be *BRCA1* methylated since our cohort was selected for familial breast cancers where *BRCA1* methylation was not infrequent^18^. Consequently, we examined the relationship between *BRCA1* methylation and cyclin E1 protein expression by interrogating the breast cancer dataset of the TCGA. 241 cases had available data for *BRCA1* methylation and cyclin E1 protein expression. Using a cut-off of 0.2 for methylation^19^, we found that *BRCA1* methylation had a significant positive correlation with cyclin E1 protein expression (*r* = 0.647, *p* = 0.0082) (Fig. 1c).

Next, we examined the association between cyclin E1 expression and overall survival in germline *BRCA1* mutated breast cancers in our cohort. High cyclin E1 expression was associated with a significantly reduced overall survival of patients with *BRCA1* mutation (233 vs 426 months, *P* = 0.012, HR 0.39, CI 0.162–0.951) (Fig. 1d).

### *CCNE1* amplification is not the primary driver of high expression of cyclin E1 in *BRCA1* mutated cancers

The *CCNE1* gene, located at chromosome position 19q12, is a frequent site of amplification in cancer. We assessed *CCNE1* amplification by in situ hybridization (ISH) analysis of tissue sections with a 19q12 probe and chromosome 19 control insulin receptor (INSR) probe to determine the 19q12/INSR ratio. Representative images of 19q12 non-amplified and 19q12 amplified tumors are shown in Fig. 1e. 13.1% (31/237) of tumors in the entire cohort were found to be 19q12 (*CCNE1)* amplified. The correlation between cyclin E1 protein and *CCNE1* gene amplification was poor (*r* = 0.178, *P* = 0.006, Fig. 1f).

Next, we assessed whether *CCNE1* amplification and *BRCA1* mutation co-occurred. In contrast to HGSOC where they do not co-occur^13^, 22/103 (21.4%) of *BRCA1* mutant cases had concurrent 19q12 (*CCNE1*) amplification (Supplementary Table 2). This was higher than *BRCA1* wildtype cases, where only 9/134 (6.7%) had 19q12 (*CCNE1*) amplification (Fig. 1g). Since 19q12 (*CCNE1*) amplification is associated with poor survival in other cancer types we examined its relationship with overall survival in *BRCA1* mutated breast cancer. Unlike high expression of cyclin E1, which is predictive of poor survival, 19q12 (*CCNE1)* amplification had no prognostic value for overall survival in *BRCA1* mutated breast cancer (Fig. 1h).

### The cyclin E1 degradation machinery is disrupted in *BRCA1* mutated breast cancers

**Fig. 2:**
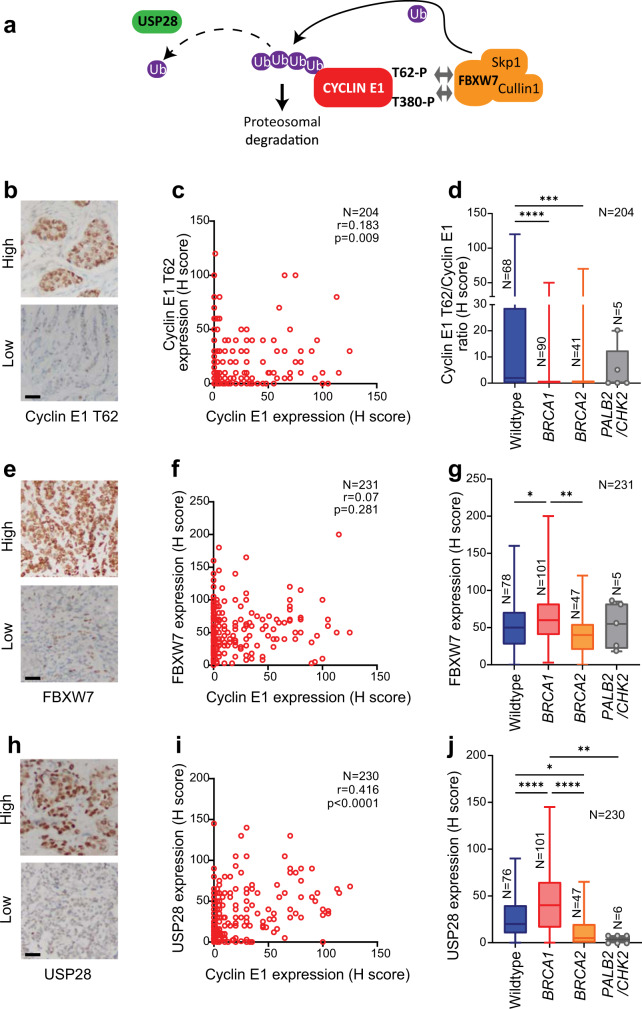
Loss of cyclin E1 T62 phosphorylation and gain of USP28 are associated with *BRCA1* mutation. **a** Schematic of cyclin E1 turnover. **b** Microscope images of IHC staining of breast cancer (BC) sections with high and low phospho-cyclin E1 T62. Scale bar is 50 µm. **c** Scatter plot of cyclin E1 expression versus phospho-cyclin E1 T62 expression, *r* = Spearman coefficient. **d** Phospho-cyclin E1 T62/cyclin E1 ratio of expression in wildtype, *BRCA1* mutated, *BRCA2* mutated*, PALB2*/*CHK2* mutated subsets of the KConfab Cohort, analyzed by one-way ANOVA; ***p < 0.001; ****p < 0.0001. Box and whisker plots show error bars of minimum to maximum, where the box extends from the 25th to the 75th percentile, and the line in the middle of the box indicates the median. **e** Microscope images of IHC staining of BC sections with high and low FBXW7. Scale bar is 50 µm. **f** Scatter plot of cyclin E1 expression versus FBXW7 expression, *r* = Spearman coefficient. **g** FBXW7 expression in wildtype, *BRCA1* mutated, *BRCA2* mutated*, PALB2*/*CHK2* mutated subsets of the KConfab Cohort, analyzed by one-way ANOVA; *p < 0.05; **p < 0.01. Box and whisker plots show error bars of minimum to maximum, where the box extends from the 25th to the 75th percentile, and the line in the middle of the box indicates the median. **h** Microscopic images of IHC staining of BC sections with high and low USP28 expression. Scale bar is 50 µm. **i** Scatter plot of cyclin E1 expression versus USP28 expression, *r* = Spearman coefficient. **j** USP28 expression in wildtype, *BRCA1* mutated, *BRCA2* mutated*, PALB2*/*CHK2* mutated subsets of the KConfab Cohort, analyzed by one-way ANOVA; *p < 0.05, **p < 0.01, ****p < 0.0001. Box and whisker plots show error bars of minimum to maximum, where the box extends from the 25th to the 75th percentile, and the line in the middle of the box indicates the median.

Since 19q12 status was only poorly predictive of high cyclin E1 expression, we thus investigated other mechanisms that lead to high cyclin E1 expression. One possibility was disruption of the proteasome-mediated degradation of cyclin E1, which occurs frequently in cancer^20^. Normal cyclin E1 turnover depends upon phosphorylation within two phospho-degrons on the cyclin E1 protein, of which T62 and T380 are crucial phosphorylation sites. The phosphorylated protein is recognized by the FBXW7 module of the SCF^FBXW7^ complex, and ubiquitinated for degradation^14^. The deubiquitinase USP28 can remove ubiquitin from cyclin E1 and antagonize FBWX7-mediated degradation^15^ (Fig. 2a). Disruption of this process, i.e., loss of cyclin E1 phosphorylation, loss of FBXW7 or gain in USP28, would be expected to increase cyclin E1 stabilization and accumulation.

We assessed the cyclin E1 degradation machinery by IHC in our familial breast cancer cohort. We first assessed cyclin E1 T62 phosphorylation in 204 cases (representative images in Fig. 2b). We observed only a moderate positive correlation between cyclin E1 T62 phosphorylation and cyclin E1 expression (*r* = 0.183, *P* = 0.009, Spearman), indicating that a proportion of cancers had very low cyclin E1 T62 phosphorylation (Fig. 2c). Consequently, we assessed the ratio of cyclin E1 phosphorylation to its absolute expression to determine if phosphorylation was specifically dysregulated in certain subsets of patients. We found that the *BRCA1* and *BRCA2* mutant subsets exhibited a significantly lower T62/cyclin E1 ratio (Fig. 2d), indicative of a loss of cyclin E1 phosphorylation in the absence of functional *BRCA1* or *BRCA2*.

There was no correlation between cyclin E1 and FBXW7 found in our cohort (Fig. 2e, f). However, *BRCA1* mutated cancers had higher expression compared to the *BRCA2* mutant subset (Fig. 2g). In contrast, USP28 expression was moderately correlated with cyclin E1 expression (*r* = 0.416, *P* < 0.0001, Spearman) (Fig. 2h, i). USP28 protein expression was significantly higher in the *BRCA1* mutated subset (Fig. 2j).

In summary, *BRCA1* mutated breast cancers were characterized by reduced cyclin E1 T62 phosphorylation and elevated USP28 expression. Overall, these data implicate increased cyclin E1 protein stability, rather than gene amplification, as the cause for high cyclin E1 levels observed in *BRCA1* mutated breast cancer.

### *BRCA1* loss leads to cell cycle stabilization of cyclin E1

**Fig. 3:**
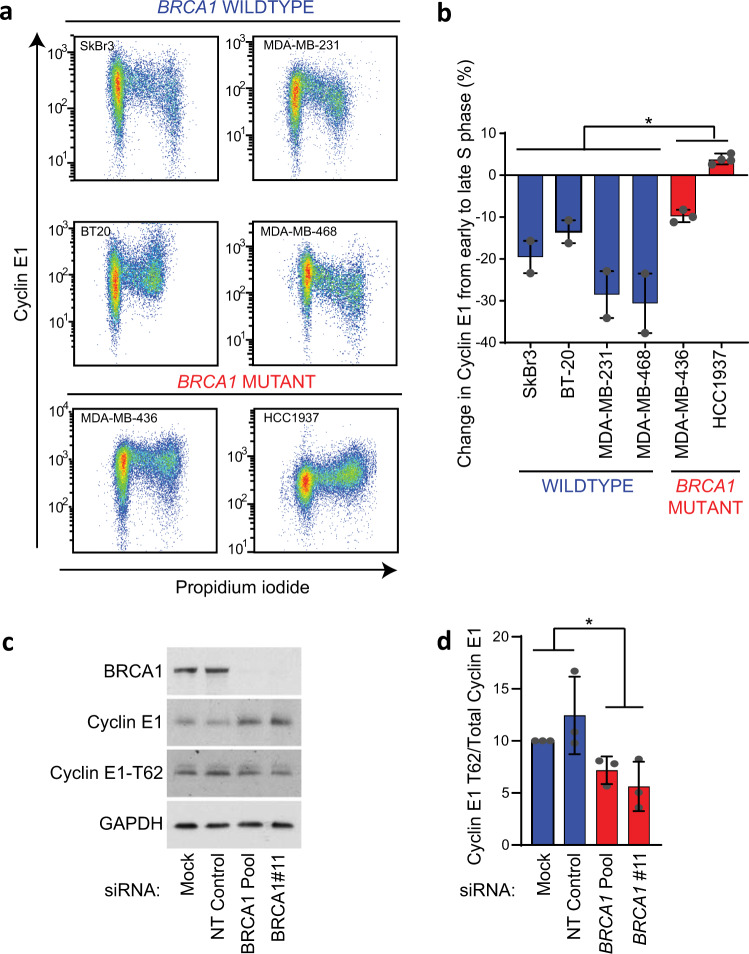
Cyclin E1 protein is stabilized in the absence of functional *BRCA1*. **a** Breast cancer cell lines (SkBr3, MDA-MB-231, BT-20, MDA-MB-468, MDA-MB-436 and HCC1937) were analyzed by flow cytometry for intracellular cyclin E1 and DNA content (propidium iodide). **b** Quantitation of fold change in expression of cyclin E1 from early S phase to late S phase as measured by flow cytometry in (**a**) of 2–4 replicates. Data are mean ± s.e.m., analyzed by *t*-test; *p = 0.05. **c** MDA-MB-468 cells were treated with siRNAs for 96 h (siRNAs: NTP (non-targeting pool) *BRCA1* #11 and *BRCA1* pool), and lysates western blotted for BRCA1, cyclin E1, cyclin E1 T62, and GAPDH. All blots derive from the same experiment and were processed in parallel. **d** MDA-MB-468 cells treated for 96 h with *BRCA1* #11 siRNA, *BRCA1* pool siRNA, and non-targeting pool siRNA were analyzed by densitometry and the ratio of cyclin E1 T62 to cyclin E1 expression determined from triplicate western blots. Data are mean ± s.e.m., analyzed by *t*-test; *p < 0.05.

Since the cyclin E1 degradation machinery was deregulated in *BRCA1* mutant cancers across our cohort, we investigated whether cyclin E1 turnover is dysregulated in cell lines with mutant *BRCA1* or *BRCA1* loss. The BLBC cell line HCC1937 has a homozygous *BRCA1* 5382C* mutation and the triple negative breast cancer (TNBC) cell line MDA-MB-436 has a *BRCA1* homozygous deletion. We compared these to 4 cell lines with wildtype *BRCA1*: BT-20 and MDA-MB-468 (BLBC cell lines), MDA-MB-231 (TNBC), and SkBr3 (HER2 amplified). Cells were analyzed for the expression of cyclin E1 during the cell cycle using flow cytometry. BT-20, MDA-MB-468, SkBr3, and MDA-MB-231 cells showed a typical downregulation of cyclin E1 during S phase (Fig. 3a), which we quantitated by comparing the expression of cyclin E1 during the second half of S phase versus the first half of S phase (Fig. 3b). The *BRCA1* defective cell lines showed significantly diminished downregulation of cyclin E1 during S phase: in HCC1937 cells the cyclin E1 levels did not decrease in S phase, but instead marginally increased. There was a small decrease in the absolute expression of cyclin E1 during S phase in MDA-MB-436 cells (Fig. 3a, b).

We next investigated whether knockdown of *BRCA1* was able to recapitulate the stabilization of cyclin E1. We treated *BRCA1* wildtype MDA-MB-468 cells, with *BRCA1* siRNA#11 and a *BRCA1* siRNA pool. Both *BRCA1* siRNAs treatments led to an increase of cyclin E1 protein (Fig. 3c). We simultaneously observed no increase in cyclin E1 T62 phosphorylation, indicating that there was lower relative phosphorylation of cyclin E1 (Fig. 3d). Thus the increased expression of cyclin E1 following depletion of BRCA1 protein was linked directly to lower phosphorylation on cyclin E1 T62.

Since cyclin E1 protein expression is dysregulated with *BRCA1* disruption and we had observed both loss of T62 phosphorylation and gain of USP28 in our cohort, we sought to confirm that *BRCA1* loss alters USP28 expression to stabilize cyclin E1. We tested whether *BRCA1* knockdown stabilized cyclin E1 via upregulation of USP28. siRNA-mediated knockdown of *USP28* in MDA-MB-231 cells led to decreased expression of cyclin E1 (Supplementary Fig. 2a). In contrast *BRCA1* siRNA led only to downregulation of BRCA1 but did not change USP28 levels (Supplementary Fig. 2b). Thus while USP28 is elevated in *BRCA1* mutant cancers, we could not detect its regulation directly downstream of *BRCA1*.

### Loss of T62 phosphorylation of cyclin E1 increases cyclin E1 stability and contributes to increased cell survival

**Fig. 4:**
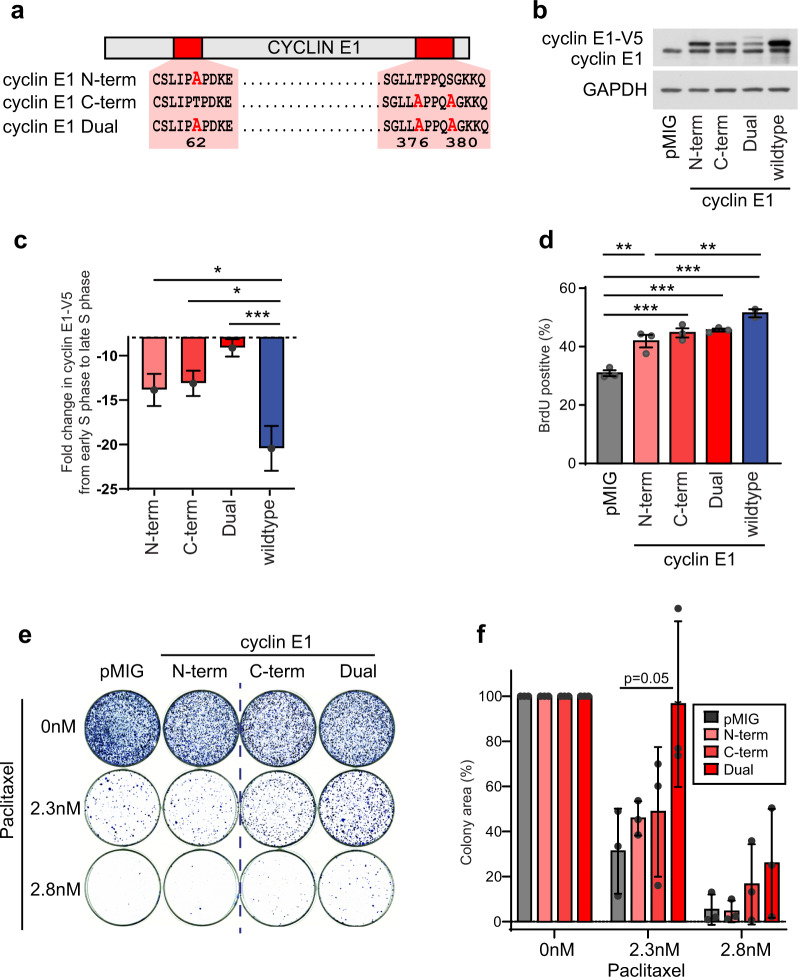
*BRCA1* loss leads to decreased cyclin E1 T62 phosphorylation, which alters protein stability, and contributes to proliferation and cell survival. **a** Schematic of site-directed mutagenesis of phospho-sites of cyclin E1. **b** MDA-MB-468 cells were retrovirally infected with V5-tagged cyclin E1 constructs (N-term, C-term, Dual, wildtype), sorted by flow cytometry for populations with matched GFP expression, and lysates western blotted for cyclin E1 and GAPDH. All blots derive from the same experiment and were processed in parallel. **c** MDA-MB-468 cells expressing cyclin E1 constructs (N-term, C-term, Dual, wildtype) were analyzed by flow cytometry for V5-cyclin E1 expression and DNA content (propidium iodide). The geometric mean expression of cyclin E1 at early S and late S phase was quantitated for each treatment, and the fold change from early to late S phase is shown as the mean ± s.e.m. Data analyzed by one-way ANOVA; *p < 0.05, ***p < 0.001. Representative experiment of triplicate experiments is shown. **d** MDA-MB-468 cells expressing cyclin E1 constructs (N-term, C-term, Dual, wildtype) were analyzed by flow cytometry for BrdU incorporation. Data are the mean ± s.e.m. of triplicate experiments or as marked, analyzed by one-way ANOVA; **p < 0.01, ***p < 0.001. **e** MDA-MB-468 cells expressing different constructs (pMIG, N-term, C-term, Dual) were treated with paclitaxel (0 nM, 2.3 nM, 2.8 nM) for 3 weeks, and colony-formation detected with Diff Quick Stain 2. **f** Colony formation was quantitated using the ColonyArea ImageJ plugin from triplicate assays. Data are the mean ± s.e.m. analyzed by one-way ANOVA at 2.3 nM paclitaxel treatment.

Since T62 dephosphorylation is associated with increased cyclin E1 protein stability, we analyzed the effect of disrupting the phospho-degrons on cyclin E1 by mutating phospho-sites to alanine to mimic the non-phosphorylated state. We performed site-directed mutagenesis within the two phospho-degrons of cyclin E1 (Fig. 4a). We created an N-terminal mutant (T62A, designated N-term), a C-terminal mutant (T376A/S380A, designated C-term), and a combined mutant (T62A/T376A/S380A, designated Dual) and stably expressed these in MDA-MB-468 BLBC cells (Fig. 4b).

We examined the effect of each mutant on the stability of the cyclin E1 protein by performing flow cytometry for the V5 tag protein during the cell cycle. We measured the fold change in each of the V5 tagged proteins between early and late S phase (Supplementary Fig. 3). All three mutants were significantly more stable than the wildtype cyclin E1 protein (Fig. 4c). Thus the T62A site in the N-terminus stabilizes the cyclin E1 protein, and particularly in combination with mutation of the C-terminal phospho-sites of cyclin E1.

Next, we examined the effect of each mutant on cell proliferation. Overexpression of cyclin E1 wildtype and each of the cyclin E1 mutants led to a significant increase in BrdU incorporation compared to the vector control (Fig. 4d).

Following this we examined whether these mutants were able to alter the survival of cells when treated with paclitaxel, a taxane chemotherapy used to treat BLBC clinically^21^. We treated vector control and cyclin E1 mutant cells with paclitaxel, and monitored survival by colony forming assay after 3–4 weeks. Only the Dual mutant cells demonstrated increased colony counts in the presence of paclitaxel compared to vector control (Fig. 4e, f).

Overall, *BRCA1* loss led to decreased cyclin E1 T62 phosphorylation, which in turn can increase cyclin E1 protein stability and percentage of cells in S phase. Cyclin E1 T62 was also critical in combination with other cyclin E1 phosphorylation sites to increase cell survival in the presence of paclitaxel.

### Synergistic targeting of cells with high cyclin E1 and *BRCA1* mutation

Our data showing that *BRCA1* loss is associated with elevated cyclin E1 protein, supporting the rationale of co-targeting these proteins. It has been demonstrated that *BRCA1* deficiency leads to susceptibility to inhibition of poly (ADP-ribose) polymerase (PARP), whereas cyclin E1 activates the therapeutically targetable kinase CDK2. However, CDK2 also has important roles in DNA repair^22^, leading to increased sensitivity of *BRCA1/2* mutant cancers to CDK2 inhibitors^23^. We thus hypothesized that treating *BRCA1* mutant cancers with a combination of CDK2 and PARP inhibitors would be synergistic due to the simultaneous blockade of cyclin E1 dependent proliferation and exacerbated synthetic lethality from PARP inhibitors due to the additional DNA damage resulting from CDK2 inhibition.

**Fig. 5:**
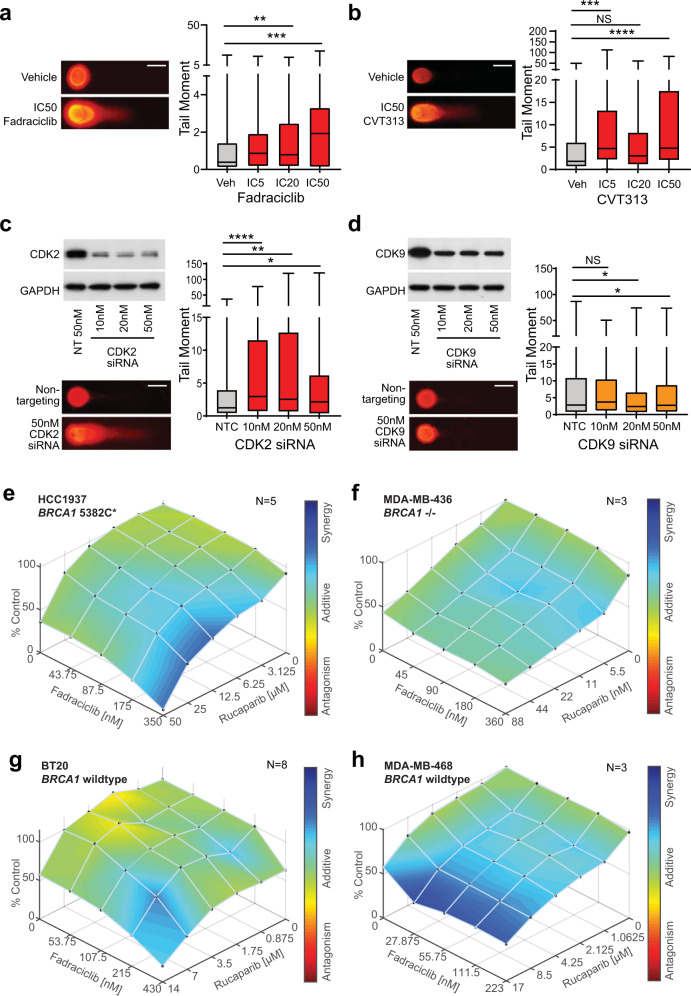
CDK2 inhibition induces DNA damage to synergize with PARP inhibition in breast cancer cells. **a** HCC1937 cells treated with fadraciclib (CYC065) or vehicle for 5 days were analyzed by alkaline Comet assay, 90–300 tails quantitated/treatment in triplicate experiments. Data analyzed by one-way ANOVA; **p < 0.01, ***p < 0.001. Representative images shown, scale bar is 50 μm. **b** HCC1937 cells treated with CVT313 or vehicle for 5 days were analyzed by alkaline Comet assay, 100–275 tails quantitated/treatment in triplicate experiments. Data analyzed by one-way ANOVA; ***p < 0.001, ****p < 0.0001. Representative images shown, scale bar is 50 μm. **c** HCC1937 cells treated with *CDK2* siRNA or Non-targeting Pool siRNA for 72 h were analyzed by alkaline Comet assay, 75–250 tails quantitated/treatment. Triplicate experiments analyzed by one-way ANOVA; *p < 0.05, **p < 0.01, ****p < 0.0001. Representative images shown, scale bar is 50 μm. Inset are western blots of matched lysates for CDK2 and GAPDH. **d** HCC1937 cells treated with *CDK9* siRNA or Non-targeting Pool siRNA for 72 h were analyzed by alkaline Comet assay, 130–300 tails quantitated/treatment. Triplicate experiments analyzed by one-way ANOVA; *p < 0.05. Representative images shown, scale bar is 50 μm. Inset are western blots of matched lysates for CDK9 and GAPDH. **e** HCC1937 cells were treated with doses of fadraciclib and rucaparib for 5 days, and viability measured by Alamar Blue. Synergy analysis was performed by BLISS, where blue indicates synergy, and red indicates antagonism. Data are pooled from 5 replicates. **f** MDA-MB-436 cells were treated with doses of fadraciclib and rucaparib for 5 days, and viability measured by Alamar Blue. Synergy analysis was performed by BLISS, where blue indicates synergy, and red indicates antagonism. Data are pooled from 3 replicates. **g** BT-20 cells were treated with doses of fadraciclib and rucaparib for 5 days, and viability measured by Alamar Blue. Synergy analysis was performed by BLISS, where blue indicates synergy, and red indicates antagonism. Data are pooled from 8 replicates. **h** MDA-MB-468 cells were treated with doses of fadraciclib and rucaparib for 5 days, and viability measured by Alamar Blue. Synergy analysis was performed by BLISS, where blue indicates synergy, and red indicates antagonism. Data are pooled from 3 replicates.

First, we tested whether CDK2 inhibition induces DNA damage, by treating *BRCA1* mutant HCC1937 cells with two CDK2 inhibitors, fadraciclib (CYC065)^24^, and CVT313^25^. We first established dose–response curves for fadraciclib and CVT313 (Supplementary Fig. 4). We then identified induction of DNA damage by both inhibitors using the alkaline Comet assay, which detects both double-strand and single-strand DNA breaks (Fig. 5a, b). While CVT313 is very specific to CDK2^25^, fadraciclib targets CDK2, CDK5, and CDK9, but with the highest specificity to CDK2^24^. CDK5 has negligible expression in HCC1937 cells^26^. We subsequently confirmed that DNA damage was occurring via CDK2 action following fadraciclib treatment by performing comet assays after treatment with *CDK2* and *CDK9* siRNA treatment. *CDK2* siRNA treatment led to an increase in tail moment detection after 72 h of exposure at all doses (Fig. 5c). In contrast, there was no effect on DNA damage following *CDK9* siRNA exposure (Fig. 5d).

Next, we examined the effect of combining CDK2 inhibition with PARP inhibition. We treated two *BRCA1* wildtype (BT-20 and MDA-MB-468) and two *BRCA1* mutant cell lines (HCC1937 and MDA-MB-436) with fadraciclib and rucaparib (a PARP inhibitor). Dose–response curves were established for each drug (Supplementary Fig. 4) and we then performed BLISS analysis to determine the effect of combining the two drugs. There was a significant synergy demonstrated with BLISS analysis between the two drugs at intersecting dose curves of fadraciclib and rucaparib. We observed additive to synergistic effects in the two *BRCA1* mutant cell lines HCC1937 and MDA-MB-436 and in *BRCA1* wildtype MDA-MB-468 cells (Fig. 5e, f, h). The *BRCA1* wildtype cell line BT-20 displayed mainly additive effects between the two drugs (Fig. 5g).

It was surprising to observe that both *BRCA1* wildtype cell lines displayed sensitivity to PARP inhibition, and MDA-MB-468 cells displayed a synergistic anti-proliferative response to combination therapy. However, MDA-MB-468 cells have been recently identified to have a *BRCA2* exon 12 deletion that leads to reduced homologous repair function^27^. When this is considered, it is not unexpected that these cells are sensitive to PARP inhibition and combination therapy. BT-20 cells have been documented to have inefficient homologous recombination pathways, which also offers a rational explanation for its partial sensitivity to PARP inhibition^28^. We thus conclude that the combination of fadraciclib and rucaparib can lead to synergistic anti-proliferative effects in cell lines with a *BRCA1* mutation, but it may also lead to significant reduction in proliferation in cell lines with other homologous recombination defects.

### Combination olaparib and fadraciclib treatment leads to tumor regression in vivo

**Fig. 6:**
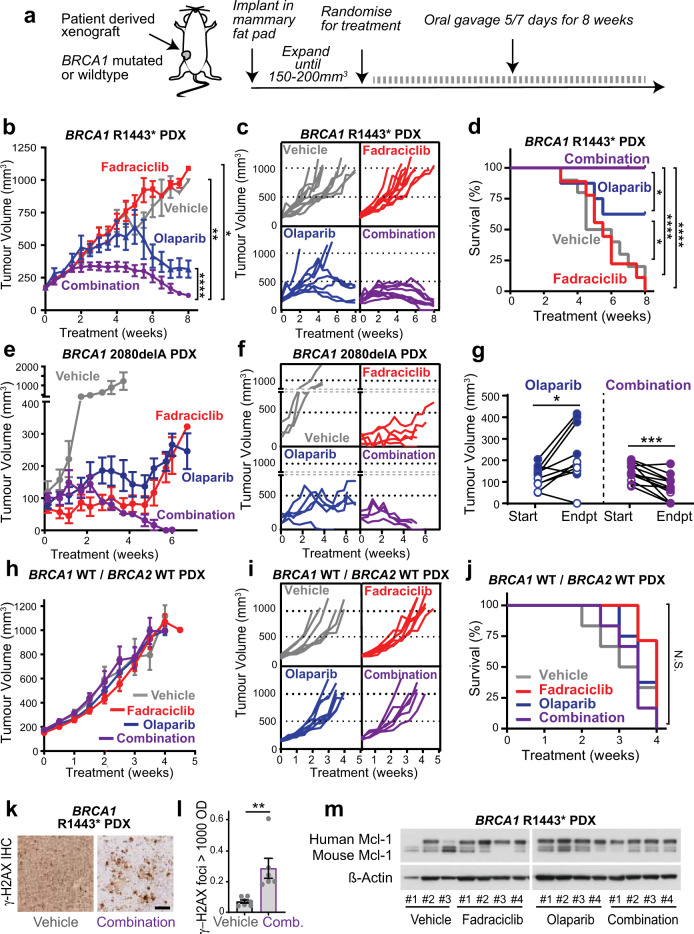
The combination of CDK2 inhibition with PARP inhibition leads to tumor regression and improved survival in *BRCA1* mutant PDX models. **a** Schematic for drug administration to BLBC PDX models. **b** BLBC *BRCA1* R1443* PDX model was treated with vehicle (gray, *n* = 10), fadraciclib (CYC065) 25 mg/kg (red, *n* = 9), olaparib 50 mg/kg (blue, *n* = 8) or the combination of fadraciclib and olaparib (purple, *n* = 9). Tumor volume was measured with calipers for 8 weeks. Data analyzed by repeated measures one-way ANOVA; *p < 0.05, **p < 0.01, ****p < 0.0001. **c** Growth kinetics of individual *BRCA1* R1443* PDX tumors with therapy. **d** Kaplan–Meier survival curves of (**b**), statistical differences between curves estimated by the Logrank (Mantel–Cox) test; *p < 0.05, ****p < 0.0001. **e** BLBC *BRCA1* 2080delA PDX model was treated with vehicle (gray, *n* = 3), fadraciclib 25 mg/kg (red, *n* = 5), olaparib 50 mg/kg (blue, *n* = 5) or the combination of fadraciclib and olaparib (purple, *n* = 4). Tumor volume was measured with calipers for 7 weeks. **f** Growth kinetics of individual *BRCA1* 2080delA PDX tumors with therapy. **g** The change in tumor volume in both *BRCA1* R1443* and *BRCA1* 2080delA PDX models between start of treatment and ethical endpoint for olaparib and combination treated cohorts. Endpoint for *BRCA1* R1443* PDX was 60 days. Endpoint for *BRCA1* 2080delA PDX was 33 days. Data analyzed by two-tailed paired *t*-test; *p < 0.05, ***p < 0.001. **h** BLBC HCI-002 (*BRCA1* WT, *BRCA2* WT) PDX was treated with vehicle (gray, *n* = 6), fadraciclib 25 mg/kg (red, *n* = 7), olaparib 50 mg/kg (blue, *n* = 8) or the combination of fadraciclib and olaparib (purple, *n* = 6). Tumor volume was measured with calipers for 5 weeks, WT = wildtype. **i** Growth kinetics of individual HCI-002 (*BRCA1* WT, *BRCA2* WT) PDX tumors with therapy, WT = wildtype. **j** Kaplan–Meier survival curves of (**h**), statistical differences between curves estimated by the Logrank (Mantel–Cox) test; N.S. = not significant. **k** γ-H2AX IHC of vehicle and combination fadraciclib and olaparib treated tumors of the *BRCA1* R1443* PDX models. Scale bar is 200 µm**. l** Quantitation of high intensity γ-H2AX foci in vehicle and combination treated tumors, analyzed by two-sided *t*-test. **m** Western blots for MCL-1 and β-Actin in lysates from vehicle (*n* = 3), fadraciclib (*n* = 4), olaparib (*n* = 4) and combination fadraciclib and olaparib (*n* = 4) treatment. All samples derive from the experiment shown in (**b**) and western blots were processed in parallel.

Since we had observed effective reduction in proliferation in *BRCA1* mutant cell lines with combination CDK2 and PARP inhibitors, we assessed the use of the combination therapy in PDX models. We tested in vivo efficacy of the combination therapy in two PDXs of BLBC origin with pathogenic *BRCA1* alterations: *BRCA1* R1443* mutation and truncating *BRCA1* 2080delA mutation. Following tumor implantation and expansion, each model was treated with daily gavage of 50 mg/kg olaparib (a PARP inhibitor) and 25 mg/kg fadraciclib (sub-optimal doses selected for combination testing) (Fig. 6a). In the *BRCA1* R1443* mutant PDX, single agent olaparib led to reduced tumor burden and increased overall survival (Fig. 6b–d). There was no single agent response to fadraciclib. By contrast, all olaparib and fadraciclib combination treated tumors regressed to below the starting tumor volume, and the host mice survived until the experimental endpoint (Fig. 6b–d).

The *BRCA1* 2080delA model has high expression of cyclin E1, probably driven in part by *CCNE1* copy number gain (1.31× from exome sequencing). In this model we found that both single agent olaparib and fadraciclib were effective, leading to a significant reduction in tumor volume. However, by the 60 day endpoint of the experiment, several of the tumors treated with either olaparib or fadraciclib had begun to grow in the presence of therapy. In contrast, the combination therapy was highly effective and resulted in tumor regression at the experimental endpoint (Fig. 6e, f).

We then further compared the effect of combination therapy to olaparib alone across the two *BRCA1* mutated models. Olaparib therapy resulted in smaller tumors compared to controls, but they were significantly larger than the starting volume (1.85× larger, *P* < 0.013, Fig. 6g). In contrast, treatment with the combination therapy led to the reduction in size of all but one tumor across the two cohorts (0.51× smaller, *P* < 0.0005; Fig. 6g).

We next examined the effect of CDK2 inhibition, PARP inhibition, and the combination therapy in a *BRCA1* wildtype model of BLBC. Since we had observed some synergy between CDK2 inhibition and PARP inhibition across in vitro models with other homologous recombination defects (Fig. 5), we chose the HCI-002 model^29^ which is wildtype for homologous recombination genes *BRCA1*, *BRCA2*, *PALB2*, *CHEK2*, and *BRIP1*^30^. We observed that the individual fadraciclib and olaparib therapies had no effect on tumor growth (Fig. 6h, i) or on survival of the animals (Fig. 6j). Moreover, the combination therapy had no effect on tumor growth or survival (Fig. 6h–j).

Finally, since fadraciclib acts via CDK2 and CDK9 inhibition, we examined the specific induction of DNA damage to determine if there was evidence for inhibition of CDK2 in the combination treated tumors. *BRCA1* R1443* PDX tumors at endpoint for vehicle showed diffuse H2AX staining across the entire tumor, whereas combination treated tumors showed intense H2AX foci and entire cells positive for H2AX (Fig. 6k), with significantly more intense staining in the combination treated tumors (Fig. 6l). We also examined a canonical marker of inhibition of CDK9, the downregulation of cell survival protein, MCL-1. MCL-1 expression was maintained or higher in fadraciclib, olaparib, and combination treated tumors than in vehicle treated tumors (Fig. 6m). Of note, fadraciclib treatment did not have any effect in the HCI-002 model (Fig. 6h–j), and this model is reported to have *CDK9* copy number gain and elevated expression^30^.

## Discussion

The PARP inhibitors olaparib and talazoparib were recently FDA approved for use as monotherapy in patients with metastatic germline *BRCA1/2*-mutated breast cancer based on significant improvement in progression-free survival compared to chemotherapy^31^. However, the utility of PARP inhibitors in BLBC is limited as clinical trials did not show an improvement in overall survival, and partial and complete responses were infrequent. Combinations with chemotherapy can be limited by myelosuppression^32^. Consequently, there is a compelling unmet clinical need to identify targeted therapies that enhance the lethality of PARP inhibitors without precipitating intolerable side-effects.

We here find that *BRCA1* loss reduces the turnover of cyclin E1 thereby increasing proliferation and survival, providing a new therapeutic opportunity to enhance the synthetic lethality of PARP inhibitors by co-targeting the cyclin E1/CDK2 axis. We evaluated fadraciclib, a CDK2/CDK5/CDK9 inhibitor which has successfully completed a First-in-Human Phase I clinical trial, and is continuing clinical development in both solid tumors and hematological malignancies. Our studies identify that CDK2 inhibitors work specifically through CDK2 to induce DNA damage in vitro. We found that a combination of CDK2 inhibition and rucaparib was efficacious across a range of in vitro models with different DNA repair deficiencies. We demonstrate high efficacy of combination fadraciclib and olaparib in vivo to induce tumor regression. Olaparib as a single agent was effective in PDX models with *BRCA1* mutation, but several individual tumors were shown to escape therapy, and overall tumor burden was increased by the experimental endpoints. In the *BRCA1* mutant PDX models, we show no tumors escaped inhibition with combination treatment, and almost all tumors regressed. We note that these in vivo models may also have benefited from the additional inhibition of CDK9 via fadraciclib, although with the conditions used we do not observe the downregulation of MCL-1 which is normally associated with CDK9 inhibition^33^ (Fig. 6m). Pan-CDK inhibitors that target CDK1 or CDK12 have been demonstrated in pre-clinical TNBC models to result in homologous repair deficiency and induce synthetic lethality in combination with PARP inhibitors^34^. This has led to the pan-CDK inhibitor dinaciclib being trialed clinically in combination with the PARP inhibitor veliparib in a patient cohort with TNBC (Clinical Trials Gov reference NCT01434316). Our data indicate that these patients may similarly derived benefit from synthetic lethality between CDK2 inhibition and PARP inhibition.

We found that cyclin E1 is stabilized in *BRCA1* mutated breast tumors in association with reduced phosphorylation on cyclin E1 Threonine 62, and high cyclin E1 is associated with decreased overall survival for patients with *BRCA1* mutation. Cyclin E1 T62 phosphorylation was originally believed to be of lesser importance in the turnover of cyclin E1, but our work here, and that of others^35^, shows that it could be having strong effects in tumorigenesis. We find that T62A mutation is sufficient to increase cyclin E1 stability and BrdU incorporation, and that T62A mutation contributes to cell survival in combination with mutation of the other major phospho-sites of the protein. Mutation of cyclin E1 T74A and cyclin E1 T393A (equivalent to human cyclin E1 T62 and T395) in a mouse model led to much higher cyclin E1 levels in hematopoietic and epithelial cells compared to T393A mutation alone, as well as hematopoietic neoplasia^35^. Delayed mammary gland involution after pregnancy was also observed exclusively in the presence of the T74A mutation^35^, highlighting its likely importance for breast tumorigenesis.

The kinase responsible for T62 phosphorylation has not been identified, though it is hypothesized to be a CDK2 auto-phosphorylation site based on a loose consensus sequence for CDK2 around the T62 site, and the timing of T62 phosphorylation early in G_1_ phase soon after partnering with CDK2^36^. Consequently, increased T62 auto-phosphorylation may be the result of a direct physical interaction between *BRCA1* and cyclin E1/CDK2^37^ or through downstream effectors of *BRCA1* action.

In summary, we have found that CDK2 inhibition may sensitize *BRCA1* mutant breast cancer cells to PARP inhibitors. *BRCA1* mutation most commonly associates with the aggressive BLBC subtype, and thus the presence of *BRCA1* mutation in concert with the BLBC phenotype would suggest combination CDK2 and PARP inhibition as an effective therapeutic strategy. As both low levels of *BRCA1* and *BRCA1* methylation are very common to BLBC^38^, and our data demonstrate elevated cyclin E1 in the *BRCA1* methylated BLBC, a rational ongoing area of investigation is CDK2 inhibition to sensitize *BRCA1* methylated or deficient cancers to PARP inhibitors.

## Methods

### Patient demographics and tumor samples

The Kathleen Cuningham Foundation Consortium for research into Familial Breast cancer (kConFab; http://www.kconfab.org) cohort comprised 308 breast cancer samples used in this analysis^39^. Ethics board approval is described in Mann et al.^39^, and was obtained for patients’ recruitment, sample collection, and research studies. Written informed consent was obtained from all participants as described^39^.

From four tissue microarrays (TMAs), 237 samples had sufficient tumor tissue for IHC. Patient and tumor characteristics are shown in Table 1. Tumors were classified based on estrogen receptor (ER), progesterone receptor (PR), HER2 status, and mutation status of *BRCA1*, *BRCA2*, *CHEK2,* and *PALB2*. CK5/14 and/or EGFR positive tumors were classified as BLBC, while CK5/14 and EGFR negative tumors were classified as non-basal like breast cancer (NBLBC).

**Table 1:** Patient and Tumour Characteristics.

AGE	MEAN	RANGE
	43.8	19 - 77
HISTOLOGICAL SUBTYPE	NUMBER	%
Infiltrating duct carcinoma, NOS	190	80.2
Carcinoma, NOS	16	6.8
Lobular carcinoma, NOS	10	4.2
Infiltrating duct and lobular carcinoma	8	3.4
Medullary carcinoma, NOS	5	2.1
Others	8	3.4
GRADE		
1	24	10.1
2	67	28.3
3	123	51.9
No grade	23	9.7
NODAL STATUS		
N0	96	40.5
N1 (1-3)	37	15.6
N2 (4-9)	13	5.5
N3 (10 or >)	6	2.5
Not known	85	35.9
GERMLINE *BRCA1/2*, *CHEK2*, *PALB2* STATUS		
*BRCA1*	103	43.5
*BRCA2*	48	20.3
*PALB2*	4	1.7
*CHEK2*	2	0.8
Wildtype	80	33.8
BASAL-LIKE CLASSIFICATION		
BLBC (CK5/CK14 and /or EGFR) Positive	76	32.1
NBLBC (CK5/14 and EGFR) Negative	136	57.4
Unclassified	25	10.5
ER/PR/HER2 STATUS		
ER/PR Positive, HER2 Negative	88	37.1
ER/PR Positive, HER2 Positive	33	13.9
ER/PR Negative, HER2 Positive	18	7.62
Triple Negative	75	31.6
Not tested	23	9.7
PROGRESSION-FREE SURVIVAL (ALL CASES)	Events	Median Months
	64	161.1
PROGRESSION-FREE SURVIVAL (*BRCA1* MUTANT)	Events	Median Months
	28	165.5
OVERALL SURVIVAL (ALL CASES)	Events	Median Months
	60	165.2
OVERALL SURVIVAL (*BRCA1* MUTANT)	Events	Median Months
	26	166.4

### Dual-color in situ hybridization (ISH) assay for detection of the 19q12 locus amplification

A 19q12 DNP ISH probe that covers the coding sequences of the *CCNE1* and adjacent *URI1* genes and an INSR DIG ISH probe, a surrogate reference located on chromosome 19p13.2, were provided by Ventana Medical Systems (Tucson, AZ), and optimized for use on the Ventana ULTRA^TM^ platform as previously described^13^, and detailed in the Supplementary methods and Supplementary Fig. 5.

### Cyclin E1, FBXW7, USP28, and phospho-cyclin E1 (T62) IHC

Previously optimized cyclin E1 mouse monoclonal (1:100 dilution, HE12) (Santa Cruz Biotechnology, CA), FBXW7 rabbit monoclonal (1:25 dilution, SP-237) (Spring Bioscience, CA) and the USP28 rabbit polyclonal (1:50 dilution, HPA006778) (Sigma Aldrich) antibody staining were performed using the Ventana Bench Mark ULTRA^TM^ automated staining platform and the Optiview^TM^ Detection kit^13^. Optimization of the cyclin E1 T62 antibody is described in the Supplementary methods and Supplementary Fig. 6. All proteins were assessed on nuclear staining using a 0 to 3+ intensity score. Heterogeneous expression was captured using the semi-quantitative H score^40^. The distribution of H scores for cyclin E1, phospho-cyclin E1 T62, FBXW7 and USP28 is shown in Supplementary Fig. 1, and determination of the assay cut points for each marker is detailed in Supplementary methods.

### Survival analysis

Kaplan–Meier curves were used to plot overall survival. Assessment of progression-free survival was not possible as progression coincided with death in many cases.

### Cell lines and drug treatment

Cell lines were obtained from ATCC and cultured in RPMI1640, 5–10% fetal calf serum and insulin (10 μg/ml). All cell lines were authenticated by STR profiling (CellBank Australia) and cultured for less than 6 months after authentication. Cyclin E1 was mutated by site-directed mutagenesis as described^41^. MDA-MB-468 cells expressing the ecotropic receptor^42^ were infected with pMSCV-IRES-GFP retrovirus expressing cyclin E1 wildtype and mutants as described^43^. Subpopulations with graded expression of GFP and cyclin proteins were separated by sterile flow cytometry and matched populations selected based on GFP expression. We expanded cell populations expressing a similar intensity of GFP signal from each cell line, and confirmed expression using western blotting.

Cells were treated with the following drugs resuspended in DSMO: rucaparib (Selleck), paclitaxel (Selleck). Fadraciclib (CYC065) was provided by Cyclacel Ltd, Dundee, UK.

### Cell proliferation and survival analysis

Survival assays were performed on MDA-MB-468 cells set up at 15,000 per 6 cm dish in 50% conditioned medium. Paclitaxel (0, 2.3, 2.8 nM) was added every 6–7 days for 3 weeks. Colonies were fixed with trichloroacetic acid (16%), and stained with 10% Diff Quik Stain 2 (Lab Aids). Quantification was done with ImageJ and the ColonyArea plugin^44^.

Metabolic rate was assessed by Alamar Blue (Invitrogen) to determine IC_50_ doses. Synergy assays were performed on indicated cell lines in 96 well plates. The concentration of each drug was increased linearly along each axis of the plate, creating a drug matrix of the different concentrations. The highest concentration of each drug was IC_80_, followed by dilutions of 1/2, 1/4, 1/8, 1/16, and no drug. Cell viability was measured after five days using Alamar Blue. Drug synergy was analyzed with Combenefit using the BLISS algorithm^45^.

### siRNA transfection

Gene-specific siRNAs to *BRCA1* (On-Target Plus siRNAs J-003461-11-0005 and J-003461-12-0005); *CDK2* (J-003236-11, J-003236-12, J-003236-13, J-003236-14); *CDK9* (J-003243-9-0002, J-003243-10-0002, J-003243-11-0002, J-003243-12-0002) and controls [On-Target Plus siCONTROLs (D-001810-10, D-001810-1-4)]; siGENOME Nontargeting siRNA #2 (D-001210-02) were purchased from Dharmacon and transfections carried out as described previously^46^.

### Western blot analysis

Cell lysates were extracted and separated on 4–12% Bis-Tris polyacrylamide gels (Invitrogen) as described^47^. Uncropped western blots are shown in Supplementary Figs. 7 and 8.

Primary antibodies were BRCA1 (1:1000 dilution, #9010, Cell Signaling Technology), USP28 (1:1000 dilution, EPR4249(2), Abcam), CDK2 (1:500 dilution, M2, Santa Cruz), CDK9 (1:1000 dilution, #2316, Cell Signaling Technology), cyclin E1 (1:1000 dilution, HE12, Santa Cruz), cyclin E1 T62 (1:400 dilution, #4136, Cell Signaling Technology), MCL-1 (1:1000 dilution, D35A5, Cell Signaling Technology), β-actin (1:2000 dilution, AC-15; Sigma) and GAPDH (1:5000 dilution, 4300; Ambion).

### Flow cytometry

S-phase percentages were measured by flow cytometric analysis of propidium iodide stained, ethanol fixed cells. Cell cycle specific expression of endogenous cyclin E1 and V5-tagged cyclin E1 constructs were assessed by flow cytometry as described^48^, with further details provided in the Supplementary Data and Supplementary Fig. 9.

### Comet assay

The alkaline comet assay was performed using the Trevigen Kit (Maryland, USA) according to the manufacturer’s guidelines. HCC1937 cells were seeded in a 6 well plate and treated with fadraciclib (Cyclacel) or CVT313 (Thermofisher) at the calculated IC_5_, IC_20_ or IC_50_ dose for 5 days, or treated with 10, 20, or 50 nM *CDK2* siRNA or *CDK9* siRNA, or 50 nM non targeting siRNA for 72 h. Slides were imaged with a fluorescence microscope (Leica DM5500) and analyzed with ImageJ OpenComet software (v1.3.1,^49^).

### TCGA datasets

Breast cancer datasets were downloaded via cBioPortal^9^ and the BLBC subset identified from PAM50 definitions from TCGA^50^.

### Patient-derived breast cancer xenograft (PDX) models

All in vivo experiments, procedures and endpoints were approved by the Garvan Institute of Medical Research Animal Ethics Committee (protocol 18/26) or the VHIO Animal Ethics Committee (protocol 17/42). PDX *BRCA1* R1443* (PDX 11-26) was derived from a metastatic triple negative breast cancer^34^, and PDX (HCI-002)^29^ and *BRCA1* 2080delA (PDX124) are basal on PAM50 classification and ER/PR negative^30,51^. For PDX (HCI-002) and PDX *BRCA1* R1443* (PDX 11-26), 3–4 mm^3^ sections of tumor tissue were implanted at surgery into the 4th inguinal mammary gland of female NOD-SCID-IL2γR^−/−^ (NSG) mice. PDX *BRCA1* 2080delA animals were implanted subcutaneously and supplemented with 1 μmol/L estradiol (Sigma) in the drinking water. Tumor growth was assessed twice weekly by caliper measurement and mice were randomized to treatment arms when tumors reached 150–250 mm^3^ (using the formula: width^2^ × length × 0.5). Vehicle (4% DMSO, 30% PEG-300), 50 mg/kg olaparib and 25 mg/kg fadraciclib were administered by oral gavage 5 days a week. Mice were treated for 60 days or until tumor volume reached 1000 mm^3^.

### PDX IHC and quantification

Tumor tissue was fixed in 10% neutral buffered formalin and embedded in paraffin, before being sectioned (4 μM thick) and stained using the Bond RX Automated Stainer (Leica Biosystems). Heat induced antigen retrieval was performed at pH 9 (Bond Epitope Retrieval solution 2, Leica Biosystems), 100 °C for 30 min, before Υ-H2AX antibody incubation (1:500 dilution, Cell Signaling, Clone 20E3) for 60 min. Detection was performed with diaminobenzidine (Bond Polymer Refine Detection, Leica Biosystems) and slides were counterstained with haematoxylin. Slides were imaged using a slide scanner (AperioCS2, Leica Biosystems), and data were analyzed using QuPath^52^ as described^53^.

### Statistical analysis

Statistical analysis was performed using Prism Software^TM^ version 7 as indicated for each dataset. Data with greater than 10 data points are presented as box and whisker plots with error bars of minimum to maximum, where the box extends from the 25th to the 75th percentile, and the line in the middle of the box indicates the median, or as bar plots with ± standard error of the mean (s.e.m.). Treatment arms and patient subgroups with less than 10 data points are presented as scatter plots without bars or with bars that indicate the mean, and error bars indicate ± s.e.m. All experiments were performed in triplicate, except as indicated. Replicates of in vitro and in vivo experiments were performed on independent samples.

### Reporting summary

Further information on research design is available in the Nature Research Reporting Summary linked to this article.

## Supplementary information

Supplementary Material

Reporting summary

## Data Availability

The data generated and analyzed during this study are described in the following data record: 10.6084/m9.figshare.14994372^54^. Where possible (and not including sensitive/patient-identifying data), the data underlying the claims of the article have been made openly available in.xlsx spreadsheet format as part of the above data record. The patient dataset is not publicly available in order to protect patient confidentiality. The clinical cohort was collected and managed by kConFab, who will consider applications to access the cohort via www.kConFab.org. Processed patient data (Excel & Prism formats) can be accessed upon reasonable enquiry with the corresponding author. Cell lines and vectors engineered by the authors for this study are available upon reasonable request to the corresponding author. PDX models were provided by A/Prof Alex Swarbrick (HCI-002), E.L. (PDX 11-26) and V.S. (PDX124).
