# Supplementary material for: Synergistic targeting of BRCA1 mutated breast cancers with PARP and CDK2 inhibition
Source: NPJ Breast Cancer. 2021 Aug 31;7:111. doi: 10.1038/s41523-021-00312-x (PMC8408175; doi:10.1038/s41523-021-00312-x)
Supplement: Supplementary file 1 — Supplementary Material [file 41523_2021_312_MOESM1_ESM.docx]

**SUPPLEMENTARY INFORMATION**

***in situ* hybridization and cut point determination**

4μm sections from formalin-fixed, paraffin-embedded TMAs were deparaffinized, treated with Cell Conditioning 2 for four cycles of 12 minutes each, followed by ISH protease 3 for 24 min. After co-denaturation, 19q12 DNP and INSR DIG probes were hybridized at 47 °C for 6 hours and washed for 3 cycles at 68 °C for 8 minutes. 19q12 DNP and INSR DIG signals were detected using VENTANA ultraView SISH DNP and VENTANA ultraView RED ISH DIG detection kits, respectively, and counterstained with hematoxylin II and bluing reagent. For image display, the brightness and contrast of each image was adjusted using Photoshop (CS6). 19q12/INSR ratio ≥ 3 and/or 19q12 average number ≥6 were deemed as amplified ^1^.

Cut-off thresholds for 19q12 amplification were guided by our previously optimised 19q12 ISH assay ^1^. Amplification status was calculated as 19q12/INSR ratio ≥ 3 and/or 19q12 average number ≥6. The distributions of 19q12/INSR ratio and 19q12 average number are provided in supplementary Figure 5A and 5B respectively.

**T62 antibody validation**

The phospho cyclin E1 (T62) polyclonal antibody (Cell Signaling) was the only IHC grade phospho-cyclin E1 antibody available, and was used at 1:50 dilution. As phospho-epitopes are potentially labile, we confirmed that cyclin E1 T62 was detectable in formalin fixed paraffin embedded tissue with a range of fixation conditions. We used paraffin embedded cell blocks of HCT116 colorectal cancer cell lines that were variably fixed in formalin at variable conditions. Different fixation conditions included both variable time to fixation, between pellet stage and 4% paraformaldehyde (PFA) addition; and variable time of fixation, the duration for which the pellet was fixed in 4% PFA. Sections from all variably formalin fixed paraffin embedded cell blocks were stained using the optimized protocol and revealed acceptable robustness of detection (Supplementary Figure 6A).

To further assess the reliability of the p T62 antibody, we tested specificity of the T62 antibody detection by examining cyclin E1 siRNA treated MDA-MB-436 cells. Western blot revealed that cyclin E1 and cyclin E1 T62 expression is lower in cyclin E1 siRNA knocked down cells, which should lead to reduced cyclin E1 T62 expression. Sections from formalin fixed paraffin embedded blocks of each of the cyclin E1 siRNA and control treated cells were stained with the optimized protocol and revealed aligned protein expression to those seen in western blot (Supplementary Figure 6B).

As those experiments revealed the stability, reliability and specificity of T62 antibody, the antibody was next used to stain sections from TMAs of samples of patients enrolled in the KConfab cohort.

**H score and cut point determination for immunohistochemistry**

The H-score was calculated by adding 3 x % of strongly staining (3+) nuclei to 2 x % of moderately staining (2+) nuclei and 1 x % of weakly staining (1+) nuclei, giving a range of 0 to 300. The overall distribution of H score of cyclin E1 expression in all cases (N=237), ranged between 0 and 125 with a median of 3 (Supplementary Figure 1A). The cutoff between high and low cyclin E1 H score was determined by two factors: (1) the previously reported frequency of high cyclin E1 expression in breast cancer and (2) the best association with outcome (minimal p value, Supplementary Table 1), leading to the selection of an H score cut-off of 45. The distribution of mean H scores for phospho-cyclin E1 T62, FBXW7 and USP28 are shown in Supplementary Figure 1B, 1C and 1D respectively. The median H scores were 5 (range: 0 - 120) for cyclin E1 T62 (N=204), 50 (range: 0 - 200) for FBXW7 (N=231) and 20 (range: 0 - 145) for USP28 (N=230). Exhaustion of tissue cores led to unequal numbers of assessable cases for each antibody.

**Flow cytometry for cell cycle specific expression of cyclin E1 and V5**

Cells were incubated overnight at 4°C with 1:100 dilutions of antibodies to the E-cyclins (E1: EP435E (Epitomics) or V5 (Invitrogen)) followed by 1 h incubation at room temperature with secondary antibodies (1:40 dilution, allophycocyanin conjugated goat anti-mouse, fluorescein 5-isothiocyanate conjugated goat anti-rabbit, Jackson Immunoresearch), co-stained with 10μg/mL PI (Sigma) for 2–5h, and incubated with 50μg/mL RNase A (Sigma). Flow cytometry was performed on a FACSCanto II (BD Biosciences). Data were analyzed using FlowJo ^2^, with gating strategy shown in Supplementary Figure 9. Cells were separated into early and late S phase by identifying the G_1_ and G_2_/M peaks and then partitioning the intervening S phase. Each S phase partition was analysed for expression of cyclin E1 or the V5 tag, where signal intensity was calculated by obtaining the geometric mean signal per cell in gated regions ^3,4^. Cyclin E1 turnover was calculated as the ratio of expression of late S phase/early S phase. V5-tagged protein turnover was calculated as the ratio of expression of late S phase/early S phase normalized to V5 expression in the pMIG control cell line.

**Western Blots**

Each panel of blots derive from the same experiment and were processed in parallel. When western blot images were altered for brightness and contrast using Photoshop to improve clarity of the image, the image was altered in its entirety. Uncropped images of blots and molecular weight markers are shown in Supplementary Figures 7 and 8.

**SUPPLEMENTARY REFERENCES**

1. Aziz, D.*, et al.* 19q12 amplified and non-amplified subsets of high grade serous ovarian cancer with overexpression of cyclin E1 differ in their molecular drivers and clinical outcomes. *Gynecologic oncology* **151**, 327-336 (2018).

2. FlowJo. (Tree Star Inc., Ashland, OR, USA, 2008).

3. Ormerod, M.G. Flow Cytometry - A Basic Introduction. (De Novo Software, Los Angeles, CA, 2008).

4. *Flow Cytometry: Principles and Applications*, (Humana Press, Totowa, New Jersey, 2007).

**
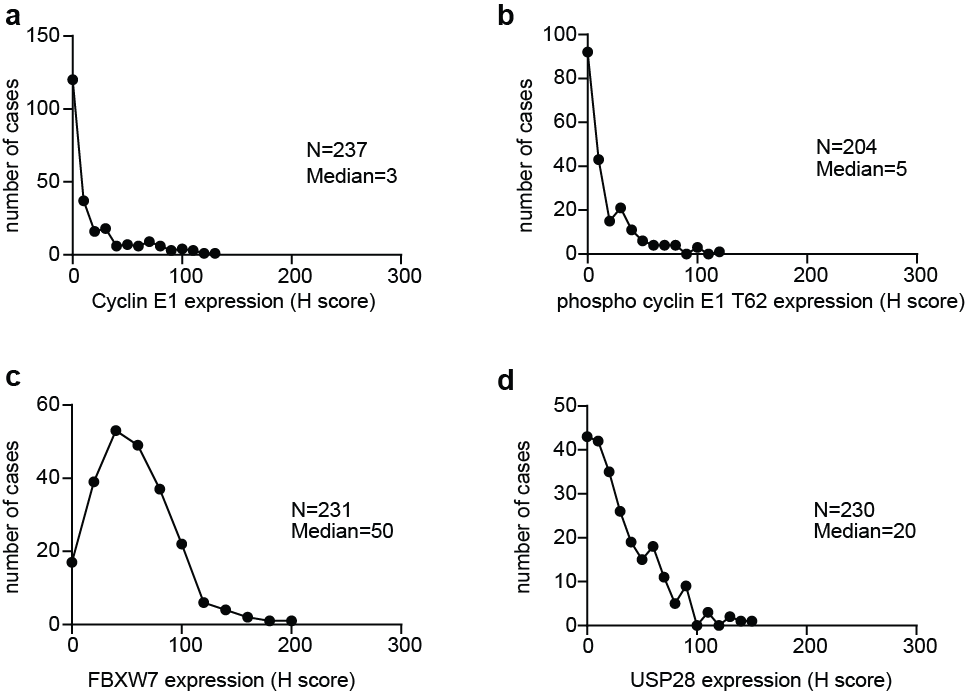
**

**Supplementary Figure 1**:  **Distribution of IHC H scores in breast cancer cases**

**A:** The distribution of IHC H scores for cyclin E1 expression in all breast cancer cases. **B:** The distribution of H scores for cyclin E1 T62 expression. **C:** The distribution of H scores for FBXW7 expression. **D:** The distribution of H scores for USP28 expression.

**
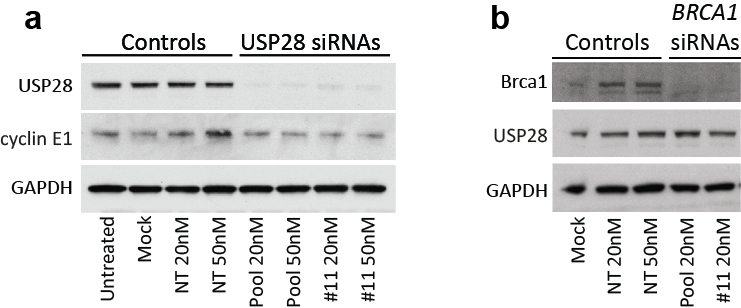
**

**Supplementary Figure 2: *BRCA1* siRNA does not alter expression of USP28**

**A:** USP28 siRNA was transfected into MDA-MB-468 cells, and lysates collected after 48h and western blotted for USP28, cyclin E1 and GAPDH. **B:** *BRCA1* siRNA was transfected into MDA-MB-468 cells, and lysates collected after 48h and western blotted for Brca1, USP28 and GAPDH. Western blots from each experiment were processed in parallel.

**
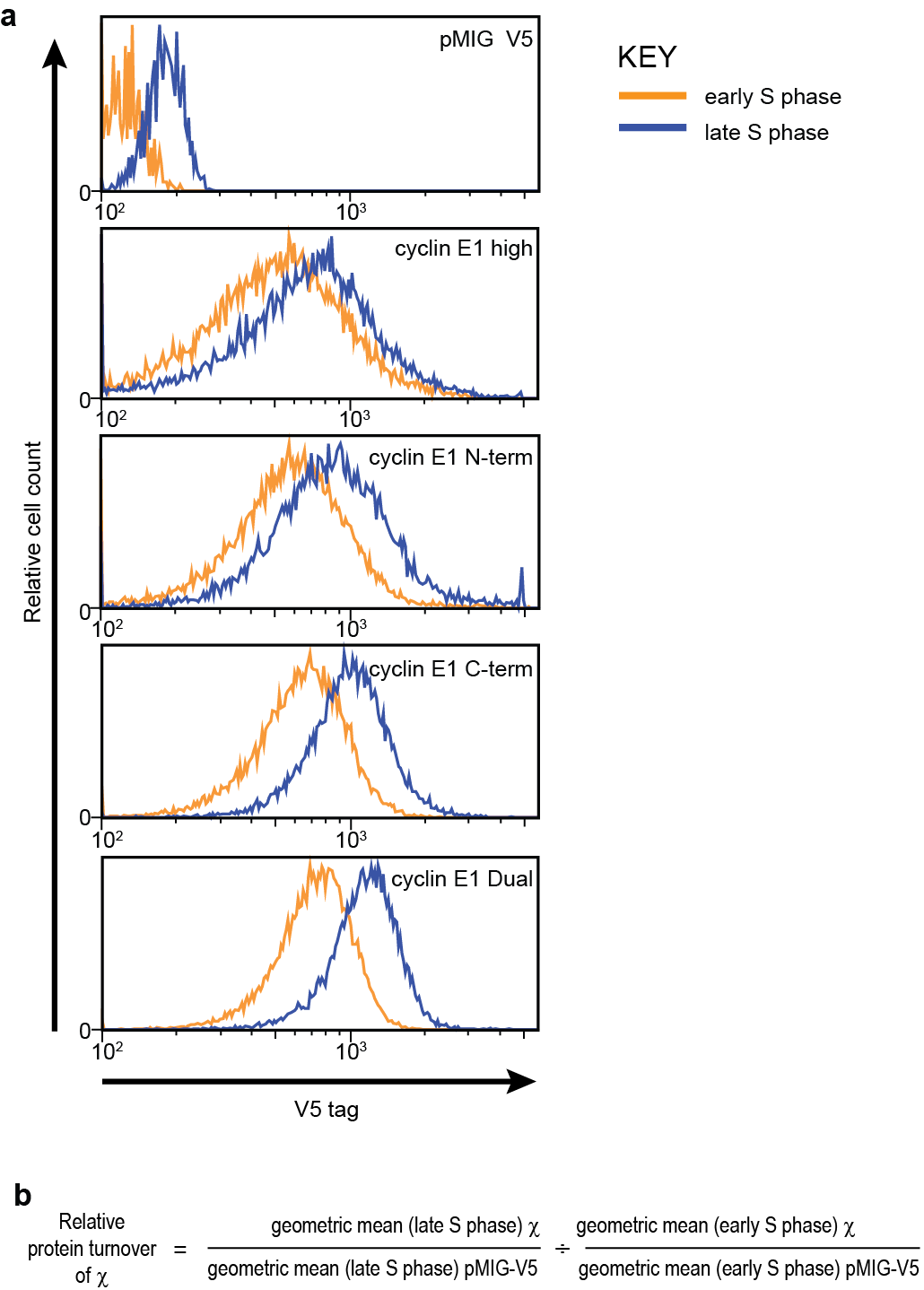
**

**Supplementary Figure 3:** **Stability of cyclin E1 constructs in S phase**

**A:** Cells expressing each of the cyclin E1 constructs (pMIG, N-term, C-term, Dual) were analysed by flow cytometry for DNA content (propidium iodide) and V5 (using anti-V5 antibody and secondary APC-labelled antibody). Cells were partitioned into early and late S-phase using propidium iodide expression, and V5 expression is shown (x-axis). **B.** Equation to determine relative stability of cyclin E1 constructs.

**
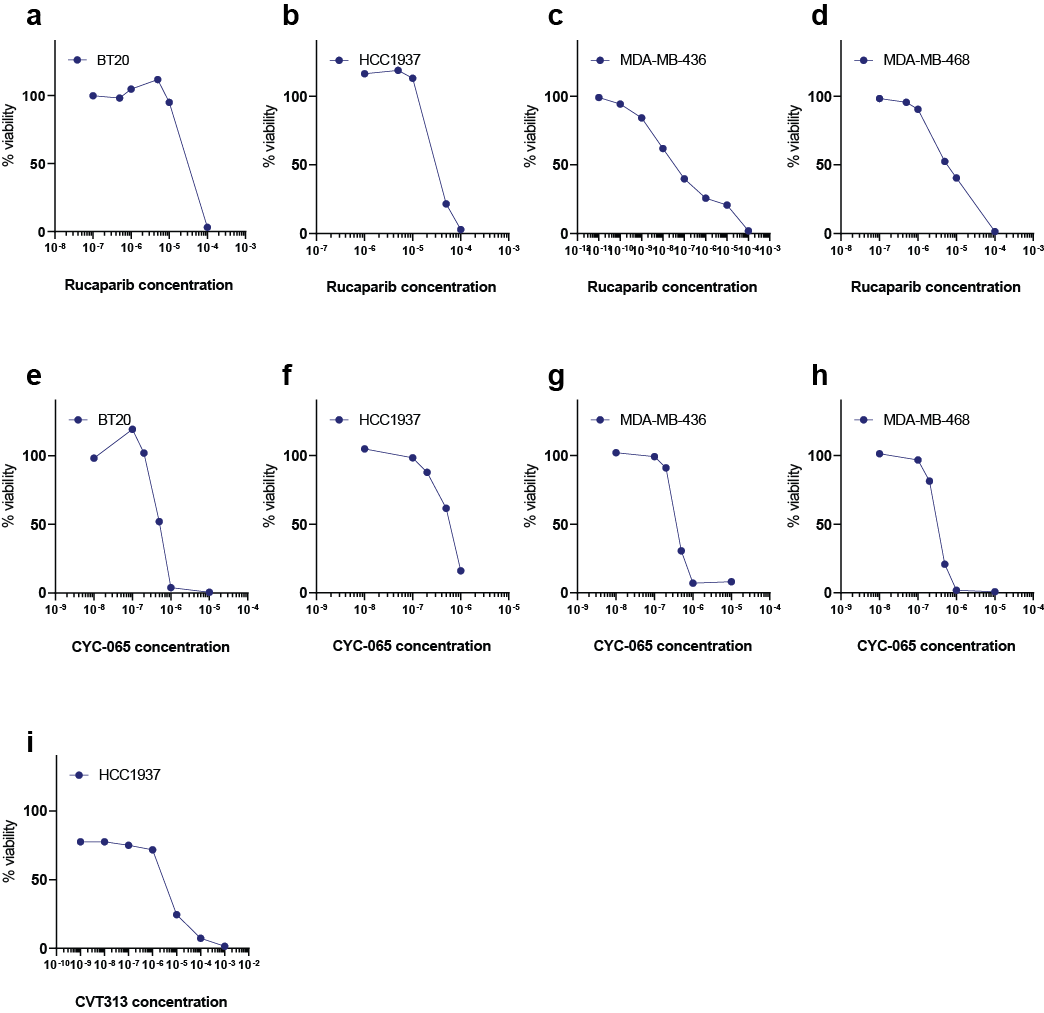
**

**Supplementary Figure 4: Dose-response curves for cell viability assessment in the BLBC cell lines BT20, HCC1937, MDA-MB-436 and MDA-MB-468.**

**A:** Cells were treated with a range of doses of rucaparib for 5 days and relative Alamar Blue staining measured. **B:** Cells were treated with a range of doses of CYC-065 for 5 days and relative Alamar Blue staining measured. **B:** Cells were treated with a range of doses of CVT313 for 5 days and relative Alamar Blue staining measured. The x-axes are molar concentration.

**
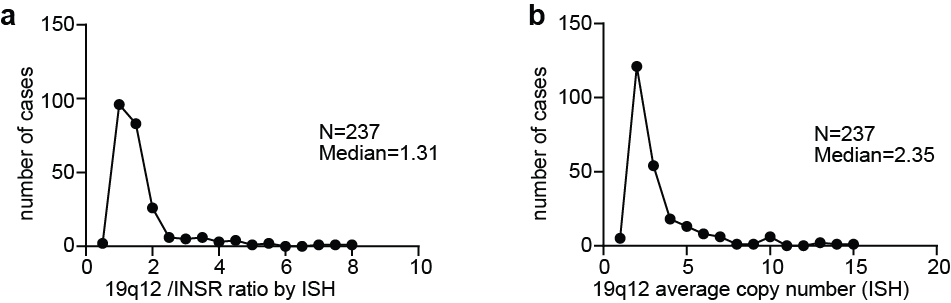
**

**Supplementary Figure 5**:  **Distribution of 19q12 ISH scores in breast cancer cases**

**A:** The distribution of 19q12/INSR ratio by ISH. **B:** The distribution of 19q12 average number.

**
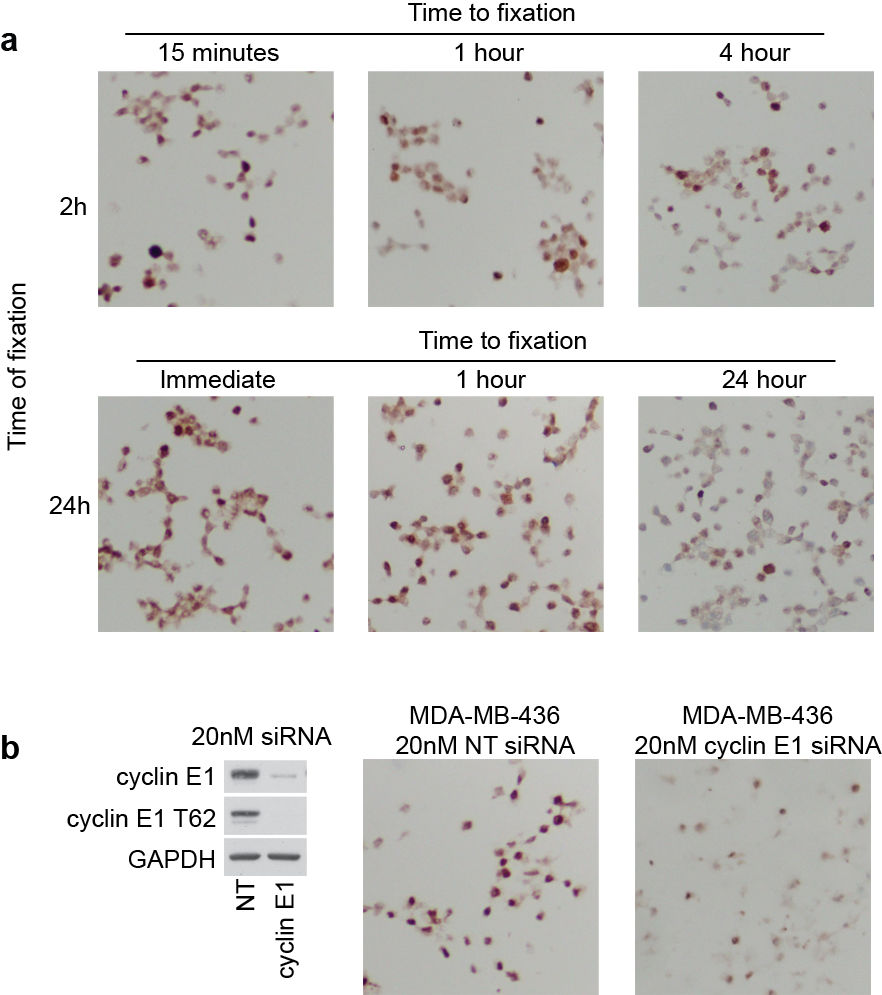
**

**Supplementary Figure 6**:  **Phospho cyclin E1 T62 antibody optimisation**

**A:** Phospho cyclin E1 T62 expression in HCT116 cell lines at different times to fixations (TTF) and time of fixation (TOF). **B:** Phospho cyclin E1 T62 expression in MDA-MB-436 cells treated with non-targeting control siRNA and cyclin E1 siRNA.

**
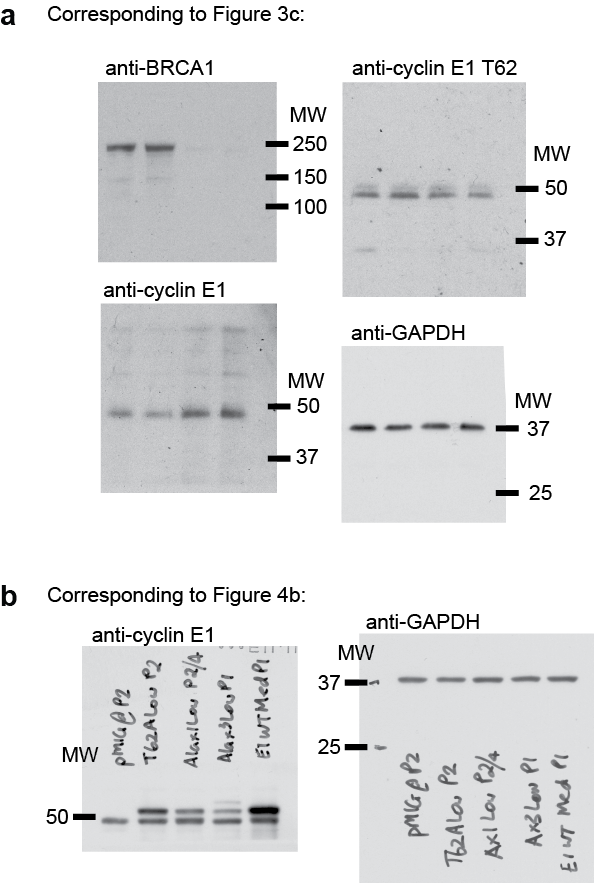
**

**Supplementary Figure 7: Uncropped western blots for Figures 3 and 4**

PVDF membranes were sliced to allow blotting for multiple primary antibodies. Shown are the uncropped western blots of the entire sliced section of PVDF membrane that correspond to **A.** Figure 3C, and **B.** Figure 4B.

**
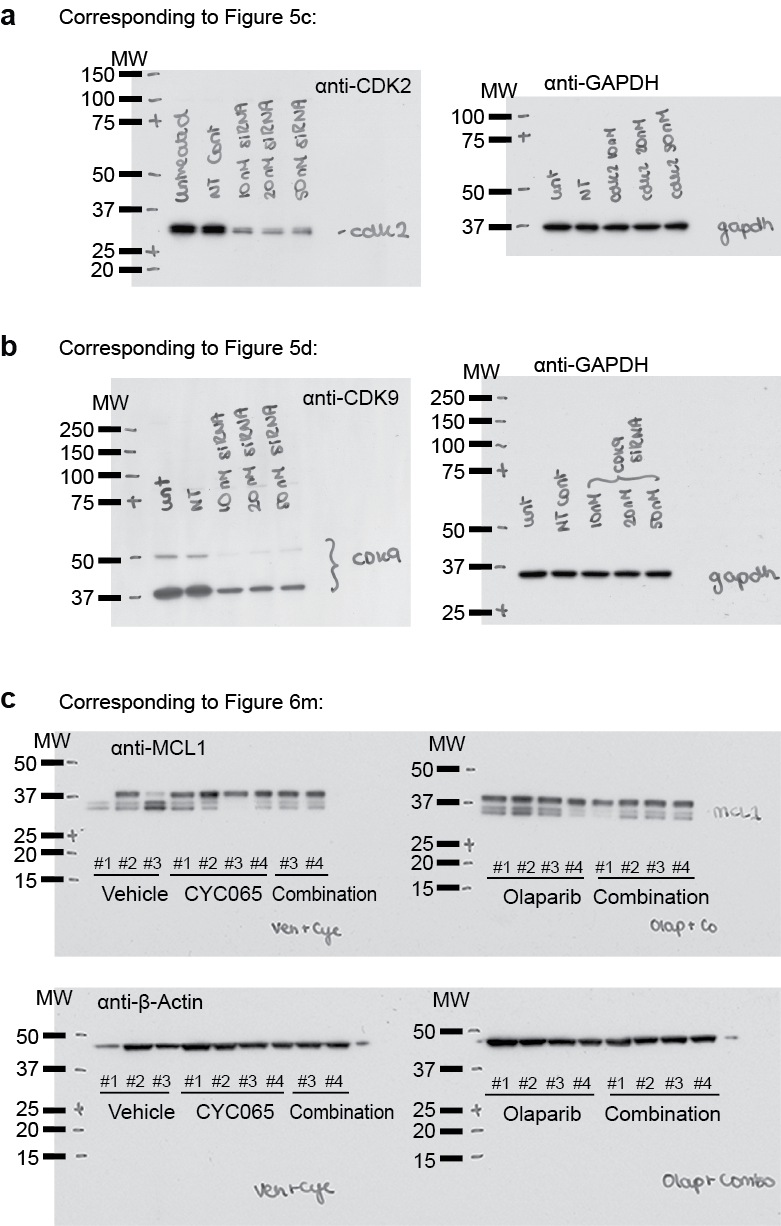
**

**Supplementary Figure 8: Uncropped western blots for Figures 5 and 6**

PVDF membranes were sliced to allow blotting for multiple primary antibodies. Shown are the uncropped western blots of the entire sliced section of PVDF membrane that correspond to **A.** Figure 5c **B.** Figure 5d and **C.** Figure 6m. In **C.** for Figure 6M, #3 and #4 combination samples were loaded on both gels/membranes to ensure calibration of signal between the western blots.

**
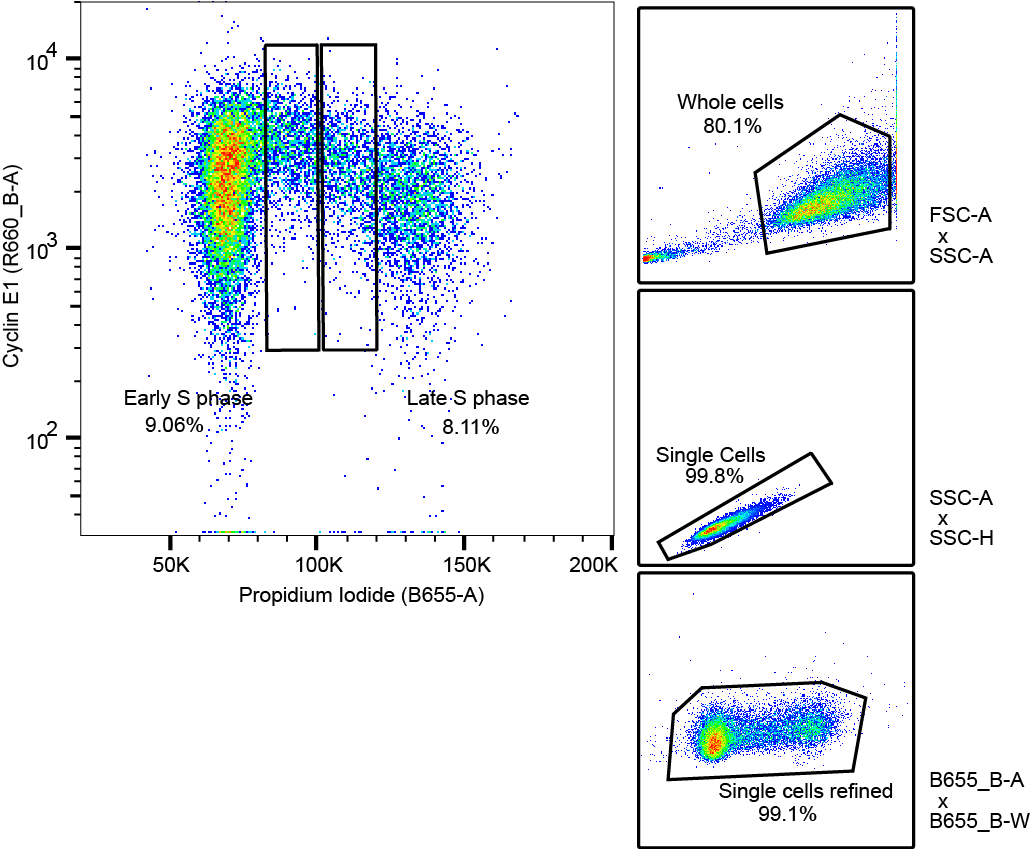
**

**Supplementary Figure 9: Gating strategy for flow cytometry**

The following gating strategy was used: FSC-A/SSC-A was used to identify and gate intact cells, SSC-A/SSC-H was used to identify and gate single cells, B575-A/B575-H was used to refine and gate single cells, Cyclin E1 expression during the cell cycle was determined using R660-B-A vs B575-A (cyclin E1 antibody) or R780-A vs B575-A (V5 antibody), and subpopulations selected for early S and late S phase.

**Supplementary Table 1: Determination of cyclin E1 cut-off based on frequency and minimum p-value**

| **H score cut-off** | **% cyclin E1 high** | **OS P value** | **OS HR** | **CI of HR** |
| --- | --- | --- | --- | --- |
| 40 | 42.7 | 0.067 | 0.866 | 0.365-2.054 |
| 45 | 40 | 0.040 | 0.866 | 0.365-2.054 |
| 50 | 36 | 0.063 | 0.548 | 0.233-1.289 |
| 55 | 33.3 | 0.055 | 0.548 | 0.233-1.289 |
| 60/65 | 29.3 | 0.198 | 0.866 | 0.365-2.054 |
| 70 | 26.7 | 0.101 | 0.866 | 0.365-2.054 |

| **All cases (237)** | **19q12 amplified (31)** | | **19q12 non amplified (206)** | |
| --- | --- | --- | --- | --- |
|  | **Cyclin E1≥45** | **Cyclin E1<45** | **Cyclin E1≥45** | **Cyclin E1<45** |
| ***BRCA1* mutated (103)** | 9 | 13 | 24 | 57 |
| ***BRCA1* non-mutated (134)** | 2 | 7 | 5 | 120 |
| **Total** | 11 | 20 | 29 | 177 |

**Supplementary Table 2: Distribution of *BRCA1* mutated and *BRCA1* non-mutated breast cancer cases versus 19q12 amplification and cyclin E1 expression status**

**kConFab Investigators consortium members:**

David Amor ^1^, Lesley Andrews ^2^, Yoland Antill ^3^, Rosemary Balleine ^4^, Jonathan Beesley ^5^, Ian Bennett ^6^, Michael Bogwitz ^7^, Leon Botes ^8^, Meagan Brennan ^9^, Melissa Brown ^10^, Michael Buckley ^11^, Jo Burke ^12^, Phyllis Butow ^13^, Liz Caldon ^14^, Ian Campbell ^15^, Deepa Chauhan ^16^, Manisha Chauhan ^17^, Georgia Chenevix-Trench ^18^, Alice Christian ^19^, Paul Cohen ^20^, Alison Colley ^21^, Ashley Crook ^22^, James Cui ^23^, Margaret Cummings ^24^, Sarah-Jane Dawson ^25^, Anna deFazio ^26^, Martin Delatycki ^27^, Rebecca Dickson ^28^, Joanne Dixon ^29^, Ted Edkins ^30^, Stacey Edwards ^31^, Gelareh Farshid ^32^, Andrew Fellows ^33^, Georgina Fenton ^34^, Michael Field ^35^, James Flanagan ^36^, Peter Fong ^37^, Laura Forrest ^38^, Stephen Fox ^39^, Juliet French ^40^, Michael Friedlander ^41^, Clara Gaff ^42^, Mike Gattas ^43^, Peter George ^44^, Sian Greening ^45^, Marion Harris ^46^, Stewart Hart ^47^, Nick Hayward ^48^, John Hopper ^49^, Cass Hoskins ^50^, Clare Hunt ^51^, Paul James ^52^, Mark Jenkins ^53^, Alexa Kidd ^54^, Judy Kirk ^55^, Jessica Koehler ^56^, James Kollias ^57^, Sunil Lakhani ^58^, Mitchell Lawrence ^59^, Shuai Li ^60^, Geoff Lindeman ^61^, Lara Lipton ^62^, Liz Lobb ^63^, Graham Mann ^64^, Deborah Marsh ^65^, Sue Anne McLachlan ^66^, Bettina Meiser ^67^, Roger Milne ^68^, Sophie Nightingale ^69^, Shona O'Connell ^70^, Sarah O'Sullivan ^71^, David Gallego Ortega ^72^, Nick Pachter ^73^, Briony Patterson ^74^, Amy Pearn ^75^, Kelly Phillips ^76^, Ellen Pieper ^77^, Edwina Rickard ^78^, Bridget Robinson ^79^, Mona Saleh ^80^, Elizabeth Salisbury ^81^, Christobel Saunders ^82^, Jodi Saunus ^83^, Rodney Scott ^84^, Clare Scott ^85^, Adrienne Sexton ^86^, Andrew Shelling ^87^, Peter Simpson ^88^, Melissa Southey ^89^, Amanda Spurdle ^90^, Jessica Taylor ^91^, Renea Taylor ^92^, Heather Thorne ^93^, Alison Trainer ^94^, Kathy Tucker ^95^, Jane Visvader ^96^, Logan Walker ^97^, Rachael Williams ^98^, Ingrid Winship ^99^, Mary Ann Young ^100^

1. Genetic Health Services, Victoria Royal Children's Hospital, Melbourne VIC 3050
2. Hereditary Cancer Clinic, Prince of Wales Hospital, Randwick NSW 2031
3. Dept. Haem and Medical Oncology, Peter MacCallum Cancer Centre, St Andrews Place, East Melbourne VIC 3002
4. Westmead Institute for Medical Research, 176 Hawkesbury Rd, Westmead NSW 2145
5. Queensland Institute of Medical Research, Herston Road, Herston Qld 4002 Australia
6. Silverton Place, 101 Wickham Terrace, Brisbane QLD 4000
7. Familial Cancer Centre, The Royal Melbourne Hospital, Grattan Street, Parkville Victoria 3050 Australia
8. Hereditary Cancer Centre, Prince of Wales Hospital, Barker St, Randwick NSW 2031 Australia
9. NSW Breast Cancer Institute, PO Box 143 Westmead NSW 2145
10. Department of Biochemistry, University of Queensland St. Lucia QLD 4072
11. Molecular and Cytogenetics Unit, Prince of Wales Hospital, Randwick NSW 2031
12. ICON Cancer Care, 2 Melville St, Hobart TAS 7000
13. Medical Psychology Unit, Royal Prince Alfred Hospital, Camperdown NSW 2204
14. Garvan Institute of Medical Research, 370 Victoria Street, Darlinghurst NSW 2010 Australia
15. Peter MacCallum Cancer Centre, St Andrew's Place, East Melbourne VIC 3002
16. School of Psychology, Brennan McCallum (Building A18) University of Sydney 2006
17. St Vincents Hospital Cancer Genetics Clinic, The Kinghorn Cancer Centre Sydney NSW
18. Queensland Institute of Medical Research, Royal Brisbane Hospital, Herston QLD 4029
19. Genetics Department, Central Region Genetics Service, Wellington Hospital New Zealand
20. St John of God Subiaco Hospital, 12 Salvado Road Subiaco WA 6008 Australia
21. Department of Clinical Genetics, Liverpool Health Service, PO Box 103 Liverpool NSW 2170
22. Department of Clinical Genetics, Level 3E, Royal North Shore Hospital, St Leonards NSW 2065
23. Epidemiology and Preventive Medicine, Monash University, Prahan Vic 3004 Australia
24. Department of Pathology, University of Queensland Medical School, Herston NSW 4006
25. Molecular Genetics Department, Cambridge University, England
26. Dept. Gynaecological Oncology, Westmead Institute for Cancer Research, Westmead Hospital, Westmead NSW 2145
27. Clinical Genetics, Austin Health Heidelberg Repatriation Hospital, PO Box 5444, Heidelberg West Vic 3081 Australia
28. Level 2, Block 51 Royal North Shore Hospital North Shore NSW 2408
29. Central Regional Genetic Services, Wellington Hospital, Private bag 7902, Wellington, New Zealand
30. Clinical Chemistry, Princess Margret Hospital for Children, Box D184 Perth WA 6001
31. Department of Biochemistry and Molecular Biology, University of Queensland, St Lucia, Qld 4072 Australia
32. Tissue Pathology, IMVS Adelaide SA 5000
33. Molecular Diagnostic Development, Pathology Department Peter MacCallum Cancer Centre, Melbourne East Melbourne Vic 3002
34. South West Family Cancer Clinic, Liverpool Hospital, Liverpool BC NSW 1871
35. Royal North Shore Hospital, Level 2, Vindin House St Leonards NSW 2065
36. Epigenetics Unit Department of Surgery and Oncology, Imperial College London, London W12 0NN England
37. Medical Oncology Department, Regional Cancer and Blood Services, Level 1 Building 7, Auckland City Hospital 2 Park Rd. Grafton, Auckland 1023 New Zealand
38. Psychosocial Cancer Genetics Research Group, Parkville Familial Cancer Centre, 305 Grattan Street ,Melbourne Vic 3000 Australia
39. Pathology Department, Level 1, Peter MacCallum Cancer Centre, St Andrew's Place East Melbourne Vic 3002
40. School of Molecular and Microbial Sciences, University of Queensland, St Lucia Qld 4072
41. Department of Medical Oncology, Prince of Wales Hospital, Randwick NSW 2031
42. Victorian Clinical Genetics Service, Royal Melbourne Hospital, Parkville VIC 3052
43. Queensland Clinical Genetic Service, Royal Children's Hospital, Bramston Terrace, Herston QLD 4020
44. Clinical Biochemistry Unit, Canterbury Health Labs, PO Box 151 Christchurch, New Zealand
45. Illawarra Cancer Centre, Wollongong Hospital, Private Mail Bag 8808 South Coast Mail Centre NSW 2521
46. Familial Cancer Clinic, Peter MacCallum Cancer Centre, St Andrews Place East Melbourne VIC 3002
47. Breast and Ovarian Cancer Genetics, Monash Medical Centre, 871 Centre Road Bentleigh East VIC 3165
48. Queensland Institute for Medical Research, Royal Brisbane Hospital, Post Office Herston QLD 4029
49. Centre for M.E.G.A. Epidemiology, University of Melbourne, Level 1, 723 Swanston Street Carlton VIC 3010
50. Parkville Familial Cancer Centre, Peter MacCallum Cancer Centre & The Royal Melbourne Hospital, Melbourne, 3000
51. Southern Health Familial Cancer Centre, Monash Medical Centre, Special Medicine Building, 246 Clayton Rd Clayton Victoria 3168 Australia
52. Genetic Health Services, Monash Medical Centre, Clayton Vic
53. Centre for M.E.G.A. Epidemiology, The University of Melbourne 723 Swanston Street, Carlton VIC 3053
54. Clinical Genetics Departments, Central Regional Genetics Service, Wellington Hospital New Zealand
55. Familial Cancer Service, Department of Medicine, Westmead Hospital Westmead NSW 2145
56. Hereditary Cancer Clinic, Prince of Wales Hospital, Randwick NSW 2031
57. Breast Endocrine and Surgical Unit, Royal Adelaide Hospital, North Terrace SA 5000
58. UQ Centre for Clinical Research, Level 6, Building 71/918 University of Queensland, The Royal Brisbane Women's Hospital, Herston, 4029
59. Prostate Cancer Research Program, 19 Innovation Walk, Level 3, Monash University, Clayton, 3800
60. Centre for Epidemiology and Biostatistics, Melbourne School of Population and Global Health, The University of Melbourne Level 3, 207 Bouverie Street, Carlton, VIC 3053
61. Breast Cancer Laboratory, Walter and Eliza Hall Institute, PO Royal Melbourne Hospital Parkville VIC 3050
62. Medical Oncology and Clinical Haematology Unit, Western Hospital Footscray, VIC
63. Medical Psychology Research Unit, Room 332, Brennan MacCallum Building (A18), The University of Sydney, Camperdown, 2006
64. Westmead Institute for Cancer Research, Westmead Millennium Institute, Westmead NSW 2145
65. Kolling Institute of Medical Research, Royal North Shore Hospital, St Leonards NSW 2065
66. Department of Oncology, St Vincent's Hospital, 41 Victoria Parade Fitzroy VIC 3065
67. Hereditary Cancer Clinic, Prince of Wales Hospital, Randwick NSW 2031
68. Centro Nacional de Investigaciones Oncologicas C/ Melchor Fernández Almagro, 3 E-28029 Madrid, Spain
69. Western Health and Peter MacCallum Cancer Centre, St Andrew's Place East Melbourne Victoria 3002
70. Southern Health Familial Cancer Centre, Special Medicine Building 246 Clayton Road, Clayton Vic 3168
71. Genetic Services, Subiaco WA 6008 Australia
72. Garvan Institute of Medical Research, The Kinghorn Cancer Centre, 370 Victoria St Darlinghurst NSW 2010 Australia
73. Familial Cancer and Clinical Genetics, Royal Melbourne Hospital, Grattan Street Parkville VIC 3050 Australia
74. Tas Clinical Genetics Service, Royal Hobart Hospital, Tasmania 7001 Australia
75. The Gene Council, Perth, Australia PO Box 510 North Perth WA 6906 Australia
76. Department of Medical Oncology, Peter MacCallum Cancer Centre, St Andrew's Place East Melbourne VIC 3002
77. Parkville Familial Cancer Centre and Genomic Medicine, VCCC Grattan Street, Melbourne Vic 3000 Australia
78. Familial Cancer Centre, Westmead Hospital, Westmead NSW 2145
79. Oncology Service, Christchurch Hospital Christchurch, New Zealand
80. Centre for Genetic Education, Prince of Wales Hospital, Randwick NSW 2031
81. Anatomical Pathology, UNSW Prince of Wales Hospital ,Randwick, 2031 NSW
82. School of Surgery and Pathology QE11 Medical Centre M block 2nd Floor, Nedlands WA 6907
83. Breast Pathology, University of Queensland, Centre for Clinical Research, Royal Brisbane and Women’s Hospital Herston QLD 4029
84. Hunter Area Pathology Service, John Hunter Hospital, NSW 2310
85. Research Department WEHI, C/o Royal Melbourne Hospital, Parkville, 3050
86. Familial Cancer Centre, Royal Melbourne Hospital, Grattan Street, Parkville Vic 3050
87. Obstetrics and Gynaecology, University of Auckland, New Zealand
88. The University of Queensland, Building 71/918 RBWH Campus Herston Qld 4029
89. Genetic Epidemiology Laboratory, Departemnt of Pathology, University of Melbourne VIC 3010
90. Cancer Unit, Queensland Institute of Medical Research, Herston QLD 4029
91. Familial Cancer and Genetics Medicine, Royal Melbourne Hospital, 2nd Floor Grattan Street, Parkville Vic 3050 Australia
92. Cancer Program, Monash University, Rm 349, Level 3, Building 76 19 Innovation Walk, Clayton VIC 3800
93. Research Department, Peter MacCallum Cancer Centre, St Andrew's Place, East Melbourne VIC 3002
94. University of NSW, Prince of Wales Hospital, Barker Street, Randwick NSW 2031
95. Heredity Cancer Clinic, Prince of Wales Hospital, Randwick NSW 2031
96. The Walter and Eliza Hall Institute of Medical Research, Royal Melbourne Hospital, Parkville VIC 3050
97. Molecular Cancer Epidemiology Laboratory, Queensland Institute of Medical Research, P.O. Royal Brisbane Hospital, Herston Qld 4027 Australia
98. Family Cancer Clinic, St Vincent's Hospital ,Darlinghurst NSW 2010
99. Department of Genetics, Royal Melbourne Hospital, Parkville, 3050
100. The Kinghorn Cancer Centre, 370 Victoria St, Darlinghurst, 2010 NSW
