# Supplementary material for: Synergistic targeting of BRCA1 mutated breast cancers with PARP and CDK2 inhibition
Source: NPJ Breast Cancer. 2021 Aug 31;7:111. doi: 10.1038/s41523-021-00312-x (PMC8408175; doi:10.1038/s41523-021-00312-x)
Supplement: Supplementary file 2 — Reporting summary [file 41523_2021_312_MOESM2_ESM.pdf]

nature research

Corresponding author(s): C E Caldon; NPJBCANCER-00593R1

Last updated by author(s): Jul 28, 2021

Reporting Summary

Nature Research wishes to improve the reproducibility of the work that we publish. This form provides structure for consistency and transparency in reporting. For further information on Nature Research policies, see our Editorial Policies and the Editorial Policy Checklist.

Statistics

For all statistical analyses, confirm that the following items are present in the figure legend, table legend, main text, or Methods section.

n/a	Confirmed	
☐	☒	The exact sample size (*n*) for each experimental group/condition, given as a discrete number and unit of measurement
☐	☒	A statement on whether measurements were taken from distinct samples or whether the same sample was measured repeatedly
☐	☒	The statistical test(s) used AND whether they are one- or two-sided<br>*Only common tests should be described solely by name; describe more complex techniques in the Methods section.*
☐	☒	A description of all covariates tested
☐	☒	A description of any assumptions or corrections, such as tests of normality and adjustment for multiple comparisons
☐	☒	A full description of the statistical parameters including central tendency (e.g. means) or other basic estimates (e.g. regression coefficient) AND variation (e.g. standard deviation) or associated estimates of uncertainty (e.g. confidence intervals)
☐	☒	For null hypothesis testing, the test statistic (e.g. *F*, *t*, *r*) with confidence intervals, effect sizes, degrees of freedom and *P* value noted<br>*Give P values as exact values whenever suitable.*
☒	☐	For Bayesian analysis, information on the choice of priors and Markov chain Monte Carlo settings
☒	☐	For hierarchical and complex designs, identification of the appropriate level for tests and full reporting of outcomes
☐	☒	Estimates of effect sizes (e.g. Cohen's *d*, Pearson's *r*), indicating how they were calculated

Our web collection on statistics for biologists contains articles on many of the points above.

Software and code

Policy information about availability of computer code

Data collection	Flow cytometry data was collected with BD FACSDiva Software (RRID:SCR_001456).
Data analysis	Statistical analyses were performed using Excel and GraphPad Prism SoftwareTM version 7.<br>Colony forming assays were analysed with ImageJ and the ColonyArea plugin [Guzmán, C., Bagga, M., Kaur, A., Westermarck, J. & Abankwa, D. ColonyArea: An ImageJ Plugin to Automatically Quantify Colony Formation in Clonogenic Assays. PLoS ONE 9, e92444 (2014).]<br>Immunohistochemistry staining was quantified with QuPath [Bankhead, P., et al. QuPath: Open source software for digital pathology image analysis. Scientific Reports 7, 16878 (2017).]<br>Comet assays were analysed with ImageJ OpenComet software (v1.3.1) [Gyori, B.M., Venkatachalam, G., Thiagarajan, P.S., Hsu, D. & Clement, M.V. OpenComet: an automated tool for comet assay image analysis. Redox biology 2, 457-465 (2014).]<br>Drug interaction assays were analysed using ComBenefit [Di Veroli, G.Y., et al. Combenefit: an interactive platform for the analysis and visualization of drug combinations. Bioinformatics 32, 2866-2868 (2016).]<br>Flow cytometry data was analysed with FlowJo. [Tree Star Inc., Ashland, OR, USA, 2008.]

For manuscripts utilizing custom algorithms or software that are central to the research but not yet described in published literature, software must be made available to editors and reviewers. We strongly encourage code deposition in a community repository (e.g. GitHub). See the Nature Research guidelines for submitting code & software for further information.

Data

Policy information about availability of data

All manuscripts must include a data availability statement. This statement should provide the following information, where applicable:

- Accession codes, unique identifiers, or web links for publicly available datasets
- A list of figures that have associated raw data
- A description of any restrictions on data availability

The data generated and analysed during this study are described in the following data record: https://doi.org/10.6084/m9.figshare.14994372 53. Where possible (and not including sensitive/patient-identifying data), the data underlying the claims of the article have been made openly available in .xlsx spreadsheet format as part of the above data record. The patient dataset is not publicly available in order to protect patient confidentiality. The clinical cohort was collected and managed by kConFab, who will consider applications to access the cohort via www.kConFab.org. Processed patient data (Excel & Prism formats) can be accessed upon reasonable enquiry with the corresponding author. Cell lines and vectors engineered by the authors for this study are available upon reasonable request to the corresponding author. PDX models were provided by Dr Alex Swarbrick (HCI-002), E.L. (PDX 11-26) and V.S. (PDX124).

Field-specific reporting

Please select the one below that is the best fit for your research. If you are not sure, read the appropriate sections before making your selection.

☒ Life sciences ☐ Behavioural & social sciences ☐ Ecological, evolutionary & environmental sciences

For a reference copy of the document with all sections, see nature.com/documents/nr-reporting-summary-flat.pdf

Life sciences study design

All studies must disclose on these points even when the disclosure is negative.

Sample size	Power calculations were performed to determine that the number of animals and tumor samples should be sufficient to determine significant differences between the different groups.
Data exclusions	Animals in which the PDX tumor did not fall within the starting tumor volume of 150-200 mm3 were not enrolled in the treatment studies. Animals which met an ethical endpoint prior to the experimental endpoint were not included in the study.
Replication	All experiments were performed at least in triplicate. Animal experiments included 3-10 animals per treatment arm.
Randomization	PDX experiments were randomised for treatment. Once tumours reached 150-200 mm3, the mice were randomized into treatment groups based on equal tumor sizes.
Blinding	Immunohistochemistry staining was evaluated by Dr Diar Aziz in a blinded setup.

Reporting for specific materials, systems and methods

We require information from authors about some types of materials, experimental systems and methods used in many studies. Here, indicate whether each material, system or method listed is relevant to your study. If you are not sure if a list item applies to your research, read the appropriate section before selecting a response.

Materials & experimental systems

n/a	Involved in the study	
☐	☒	Antibodies
☐	☒	Eukaryotic cell lines
☒	☐	Palaeontology and archaeology
☐	☒	Animals and other organisms
☐	☒	Human research participants
☐	☒	Clinical data
☒	☐	Dual use research of concern

Methods

n/a	Involved in the study	
☒	☐	ChIP-seq
☐	☒	Flow cytometry
☒	☐	MRI-based neuroimaging

Antibodies

Antibodies used	BRCA1 (#9010, Cell Signaling Technology), USP28 (EPR4249(2), Abcam), CDK2 (M2, Santa Cruz), cyclin E1 (HE12, Santa Cruz), Mcl-1 (D35A5, Cell Signaling), β-actin (AC-15; Sigma), E1 (EP435E, Epitomics), V5 (Invitrogen) and GAPDH (4300; Ambion).
Validation	All antibodies are commercially available and have been validated by the company for Western blotting and/or immunohistochemistry. In addition:<br>- validation of the specificity of the cyclin E1-T62 antibody for immunohistochemistry is described in Supplementary Figure 6.

- validation of the specificity of the USP28, cyclin E1 and Fbxw7 antibodies for immunohistochemistry is described in (1).
- validation of the cyclin E1 antibody for flow cytometry is described in (2).

1. Aziz, D., et al. 19q12 amplified and non-amplified subsets of high grade serous ovarian cancer with overexpression of cyclin E1 differ in their molecular drivers and clinical outcomes. Gynecologic Oncology (2018).
2. Caldon, C.E., Sergio, C.M., Sutherland, R.L. & Musgrove, E.A. Differences in degradation lead to asynchronous expression of cyclin E1 and cyclin E2 in cancer cells. Cell Cycle 12, 596-605 (2013).

Eukaryotic cell lines

Policy information about cell lines

Cell line source(s)	Cell lines were obtained from ATCC.
Authentication	All cell lines were authenticated by STR profiling (CellBank Australia) and cultured for less than 6 months after authentication.
Mycoplasma contamination	Cell lines were routinely screened for mycoplasma contamination using the Lonza MycoAlert Kit.
Commonly misidentified lines (See ICLAC register)	BT-20: This cell line is derived from a basal-like breast cancer, and is appropriate for this study. It is not contaminated as it was authenticated by STR profiling.

Animals and other organisms

Policy information about studies involving animals; ARRIVE guidelines recommended for reporting animal research

Laboratory animals	Studies were performed with 8-10 week old female NOD-SCID-IL2γR-/- (NSG) mice.
Wild animals	N/A
Field-collected samples	N/A
Ethics oversight	All in vivo experiments, procedures and endpoints were approved by the Garvan Institute of Medical Research Animal Ethics Committee (protocol 18/26) or the VHIO Animal Ethics Committee (protocol 17/42).

Note that full information on the approval of the study protocol must also be provided in the manuscript.

Human research participants

Policy information about studies involving human research participants

Population characteristics	308 breast cancer samples were used from The Kathleen Cuningham Foundation Consortium for research into Familial Breast cancer (kConFab; http://www.kconfab.org) cohort. The selection of the population and recruitment are described in (1).<br>1. Mann GJ, Thorne H, Balleine RL, Butow PN, Clarke CL, Edkins E, Evans GM, Fereday S, Haan E, Gattas M, Giles GG, Goldblatt J, Hopper JL, Kirk J, Leary JA, Lindeman G, Niedermayr E, Phillips KA, Picken S, Pupo GM, Saunders C, Scott CL, Spurdle AB, Suthers G, Tucker K, Chenevix-Trench G; Kathleen Cuningham Consortium for Research in Familial Breast Cancer. Analysis of cancer risk and BRCA1 and BRCA2 mutation prevalence in the kConFab familial breast cancer resource. Breast Cancer Res. 2006;8(1):R12. doi: 10.1186/bcr1377. Epub 2006 Feb 13. PMID: 16507150; PMCID: PMC1413975.
Recruitment	Recruitment is described in (1) or at www.kConfab.org.<br>1. Mann GJ, Thorne H, Balleine RL, Butow PN, Clarke CL, Edkins E, Evans GM, Fereday S, Haan E, Gattas M, Giles GG, Goldblatt J, Hopper JL, Kirk J, Leary JA, Lindeman G, Niedermayr E, Phillips KA, Picken S, Pupo GM, Saunders C, Scott CL, Spurdle AB, Suthers G, Tucker K, Chenevix-Trench G; Kathleen Cuningham Consortium for Research in Familial Breast Cancer. Analysis of cancer risk and BRCA1 and BRCA2 mutation prevalence in the kConFab familial breast cancer resource. Breast Cancer Res. 2006;8(1):R12. doi: 10.1186/bcr1377. Epub 2006 Feb 13. PMID: 16507150; PMCID: PMC1413975.
Ethics oversight	Ethics oversight is described in (1) or at www.kConfab.org.<br>1. Mann GJ, Thorne H, Balleine RL, Butow PN, Clarke CL, Edkins E, Evans GM, Fereday S, Haan E, Gattas M, Giles GG, Goldblatt J, Hopper JL, Kirk J, Leary JA, Lindeman G, Niedermayr E, Phillips KA, Picken S, Pupo GM, Saunders C, Scott CL, Spurdle AB, Suthers G, Tucker K, Chenevix-Trench G; Kathleen Cuningham Consortium for Research in Familial Breast Cancer. Analysis of cancer risk and BRCA1 and BRCA2 mutation prevalence in the kConFab familial breast cancer resource. Breast Cancer Res. 2006;8(1):R12. doi: 10.1186/bcr1377. Epub 2006 Feb 13. PMID: 16507150; PMCID: PMC1413975.

Note that full information on the approval of the study protocol must also be provided in the manuscript.

Clinical data

Policy information about clinical studies

All manuscripts should comply with the ICMJE guidelines for publication of clinical research and a completed CONSORT checklist must be included with all submissions.

Clinical trial registration	N/A
Study protocol	N/A

Data collection	N/A
Outcomes	N/A

Flow Cytometry

Plots

Confirm that:

☒ The axis labels state the marker and fluorochrome used (e.g. CD4-FITC).

☒ The axis scales are clearly visible. Include numbers along axes only for bottom left plot of group (a 'group' is an analysis of identical markers).

☒ All plots are contour plots with outliers or pseudocolor plots.

☒ A numerical value for number of cells or percentage (with statistics) is provided.

Methodology

Sample preparation	Adherent cell cultures were trypsinised, resuspended as single cells and then fixed in methanol. Cells were permeabilised with PBS + 0.1% Triton, and then incubated sequentially with primary antibody, secondary antibody and propidium iodide co-stain as detailed in the Supplementary Methods.
Instrument	Flow cytometry was performed on a FACSCanto II (BD Biosciences).
Software	Data were analyzed using FlowJo (FlowJo. 7.2.5. Ashland, OR, USA: Tree Star Inc.; 2008).
Cell population abundance	In analysis experiments between 10,000 and 30,000 B575-A/B575-H gated cells were analysed.<br>No cell sorting was performed.
Gating strategy	In each experiment:<br>- FSC-A/SSC-A was used to identify and gate intact cells<br>- SSC-A/SSC-H was used to identify and gate single cells<br>- B575-A/B575-H was used to refine and gate single cells.<br>- Cyclin E1 expression during the cell cycle was determined using R780-A vs B575-A, and subpopulations selected for early S and late S phase.<br>Additional gating is shown in Supplementary Figure 9.

☒ Tick this box to confirm that a figure exemplifying the gating strategy is provided in the Supplementary Information.
